# DC subset–specific induction of T cell responses upon antigen uptake via Fcγ receptors in vivo

**DOI:** 10.1084/jem.20160951

**Published:** 2017-05-01

**Authors:** Christian H.K. Lehmann, Anna Baranska, Gordon F. Heidkamp, Lukas Heger, Kirsten Neubert, Jennifer J. Lühr, Alana Hoffmann, Katharina C. Reimer, Christin Brückner, Simone Beck, Michaela Seeling, Melissa Kießling, Didier Soulat, Anne B. Krug, Jeffrey V. Ravetch, Jeanette H.W. Leusen, Falk Nimmerjahn, Diana Dudziak

**Affiliations:** 1 Laboratory of Dendritic Cell Biology, Department of Dermatology, University Hospital of Erlangen, Friedrich-Alexander-Universität (FAU) Erlangen-Nürnberg, 91054 Erlangen, Germany; 2 Department of Biology, Chair of Genetics, Friedrich-Alexander-Universität (FAU) Erlangen-Nürnberg, 91054 Erlangen, Germany; 3 Mikrobiologisches Institut – Klinische Mikrobiologie, Immunologie und Hygiene, University Hospital of Erlangen, Friedrich-Alexander-Universität (FAU) Erlangen-Nürnberg, 91054 Erlangen, Germany; 4 Medical Immunology Campus Erlangen, Friedrich-Alexander-Universität (FAU) Erlangen-Nürnberg, 91054 Erlangen, Germany; 5 Institute for Immunology, Biomedical Center, Ludwig-Maximilians-University Munich, 82152 Planegg-Martinsried, Germany; 6 Leonard Wagner Laboratory of Molecular Genetics and Immunology, The Rockefeller University, New York, NY 10065; 7 Immunotherapy Laboratory, Laboratory for Translational Immunology, University Medical Center Utrecht, 3584 Utrecht, Netherlands; 8 Centre d’Immunologie de Marseille-Luminy, Aix Marseille Université, Institut National de la Santé et de la Recherche Médicale–Centre National de la Recherche Scientifique, 13288 Marseille-Luminy, France

## Abstract

Dendritic cells (DCs) are efficient antigen-presenting cells equipped with various cell surface receptors for the direct or indirect recognition of pathogenic microorganisms. Interestingly, not much is known about the specific expression pattern and function of the individual activating and inhibitory Fcγ receptors (FcγRs) on splenic DC subsets in vivo and how they contribute to the initiation of T cell responses. By targeting antigens to select activating and the inhibitory FcγR in vivo, we show that antigen uptake under steady-state conditions results in a short-term expansion of antigen-specific T cells, whereas under inflammatory conditions especially, the activating FcγRIV is able to induce superior CD4^+^ and CD8^+^ T cell responses. Of note, this effect was independent of FcγR intrinsic activating signaling pathways. Moreover, despite the expression of FcγRIV on both conventional splenic DC subsets, the induction of CD8^+^ T cell responses was largely dependent on CD11c^+^CD8^+^ DCs, whereas CD11c^+^CD8^−^ DCs were critical for priming CD4^+^ T cell responses.

## Abstract (precis)

Lehmann et al. targeted antigens to Fcγ receptors expressed on various antigen-presenting cells. Induced CD4^+^ and CD8^+^ T cell responses were solely dependent on CD11b^+^ and CD8^+^ DC subsets, respectively, but independent of receptor intrinsic ITAM or ITIM signaling domains.

## Introduction

DCs are critical for the induction of protective immune responses to pathogens as well as for the maintenance of tolerance to self- and innocuous foreign antigens (Banchereau and Steinman, 1998; Steinman et al., 2003b; Steinman and Banchereau, 2007). Therefore, DCs continuously sample their surrounding environment with different pattern recognition and endocytosis receptors such as TLRs, nucleotide oligomerization domain (NOD-like) receptors, C-type lectin receptors, and Fc receptors (Figdor et al., 2002; Edwards et al., 2003; Nimmerjahn and Ravetch, 2006, 2008; Kawai and Akira, 2011; Tacken et al., 2011b; Unger and van Kooyk, 2011; Broz and Monack, 2013; Guilliams et al., 2014; Hoving et al., 2014; Pincetic et al., 2014; Heidkamp et al., 2016b).

By binding the constant fragment of IgG, Fcγ receptors (FcγRs) are important for the recognition and clearance of IgG opsonized microorganisms by phagocytes, but they also enhance antigen uptake and presentation by DCs and macrophages (Amigorena et al., 1998; Regnault et al., 1999; Machy et al., 2000; Wernersson et al., 2000; Pooley et al., 2001; Wallace et al., 2001; den Haan and Bevan, 2002; Kalergis and Ravetch, 2002; Rafiq et al., 2002; Schuurhuis et al., 2002; Sedlik et al., 2003; Tobar et al., 2004; de Jong et al., 2006; Harbers et al., 2007; Herrada et al., 2007; Taylor et al., 2007; van Montfoort et al., 2012; Guilliams et al., 2014). To date, three different activating and one inhibitory FcγRs have been described in mice and humans, which can be distinguished by their affinity for the different IgG subclasses (Takai, 2005; Nimmerjahn and Ravetch, 2006, 2008; Powell and Hogarth, 2008; Willcocks et al., 2009). Although the inhibitory FcγRIIB (CD32b) contains an intrinsic immune receptor tyrosine-based inhibitory motif in the cytoplasmic tail, the activating receptors FcγRI (CD64), FcγRIII (CD16), and FcγRIV need to interact with the immune receptor tyrosine-based activation motif (ITAM) containing Fcε receptor γ-chain to trigger cell activation (Amigorena et al., 1992a,b; Scholl and Geha, 1993; Duchemin et al., 1994; Takai et al., 1994; Sedlik et al., 2003; Nimmerjahn et al., 2005; Herrada et al., 2007; Pincetic et al., 2014). Of note, coexpression of activating and inhibitory FcγRs was demonstrated to set a threshold for activation of innate immune effector cells and B cells (Tarasenko et al., 2007; Niederer et al., 2010; Lehmann et al., 2012). In a similar manner, both activating and inhibitory FcγRs were shown to be expressed on mouse and human monocyte–derived DCs (Regnault et al., 1999; Kalergis and Ravetch, 2002; Schuurhuis et al., 2002; Bánki et al., 2003; Sedlik et al., 2003; Tan et al., 2003; Boruchov et al., 2005; Dhodapkar et al., 2005; Nimmerjahn et al., 2005; Hartwig et al., 2010). Furthermore, it was suggested that the inhibitory FcγR may be critical for the prevention of premature human DC activation by small amounts of circulating immune complexes normally present in human plasma under steady-state conditions (Dhodapkar et al., 2005). In mice, immunization with immune complexes was reported to induce DC maturation followed by presentation of antigen-derived peptides on MHCI and MHCII (Regnault et al., 1999; Machy et al., 2000; den Haan and Bevan, 2002; Kalergis and Ravetch, 2002; Schuurhuis et al., 2002; Desai et al., 2007; Björck et al., 2008). Loading of FcγRIIB-deficient mouse bone marrow–derived DCs with immune complexes followed by their transfer into mice further enhanced the cytotoxic T cell priming (Kalergis and Ravetch, 2002). In addition to conventional DCs (cDCs), which express a variety of FcγRs (Steinman and Cohn, 1974; Steinman et al., 1979; Regnault et al., 1999; den Haan and Bevan, 2002; Tan et al., 2003; Nimmerjahn et al., 2005; Desai et al., 2007; Björck et al., 2008; Syed et al., 2009; Hartwig et al., 2010; Langlet et al., 2012; Plantinga et al., 2013), a selective FcγRIIB expression was reported on plasmacytoid DCs (pDCs; Desai et al., 2007; Flores et al., 2009). Whether FcγR-dependent uptake of immune complexes into pDCs can prime T cell responses is a matter of debate (Benitez-Ribas et al., 2006; Björck et al., 2008; Flores et al., 2009; Tel et al., 2013). Although these previous studies support the notion that FcγRs on DCs are critical for the induction and enhancement of T cell responses toward IgG opsonized antigens, they do not address how the different DC subsets localized to anatomically different regions in the spleen contribute to these responses. Indeed, previous studies suggest that the two cDC subsets, and also pDCs, may have a differential capacity to initiate either CD8^+^ or CD4^+^ T cell responses upon specific loading with antigen in vivo (Ludewig et al., 2000; Hawiger et al., 2001; den Haan and Bevan, 2002; Bonifaz et al., 2004; Dudziak et al., 2007; Soares et al., 2007; Yamazaki et al., 2008; Wei et al., 2009; Kamphorst et al., 2010; Lahoud et al., 2011; Loschko et al., 2011; Tacken et al., 2011a; Moeller et al., 2012; Neubert et al., 2014; Heidkamp et al., 2016a; Lehmann et al., 2016). Furthermore, expression of the most recently discovered activating FcγRIV, which is closely related to human FcγRIIIA and shown to be essential for IgG2a- and IgG2b-dependent effector functions, was not investigated in detail (Nimmerjahn et al., 2005; Syed et al., 2009; Aschermann et al., 2013).

To address these questions, we first characterized the expression of all activating and inhibitory FcγRs on splenic DC subsets and showed that all receptors are broadly expressed on cDCs, whereas pDCs only express FcγRIIB. By directly targeting antigens via FcγR-specific antibodies to the inhibitory FcγRIIB or the activating FcγRIV, we show that both receptors are able to induce CD4^+^ and CD8^+^ T cell responses under steady-state conditions dependent on either CD11c^+^CD8^−^ or CD11c^+^CD8^+^ DCs, which in both cases ultimately resulted in T cell number reduction. Under inflammatory conditions, however, antigens taken up via the activating FcγRIV demonstrated superior CD4^+^ and CD8^+^ T cell responses in vivo. Interestingly, these T cell responses were independent of FcγR ITAM signaling, demonstrated using NOTAM mice mutated in the ITAM of the Fcε receptor γ-chain (de Haij et al., 2010; Boross et al., 2014).

## Results

### Expression of FcγRs on splenic DCs and characterization of antigen uptake via FcγRs

**Figure 1.**
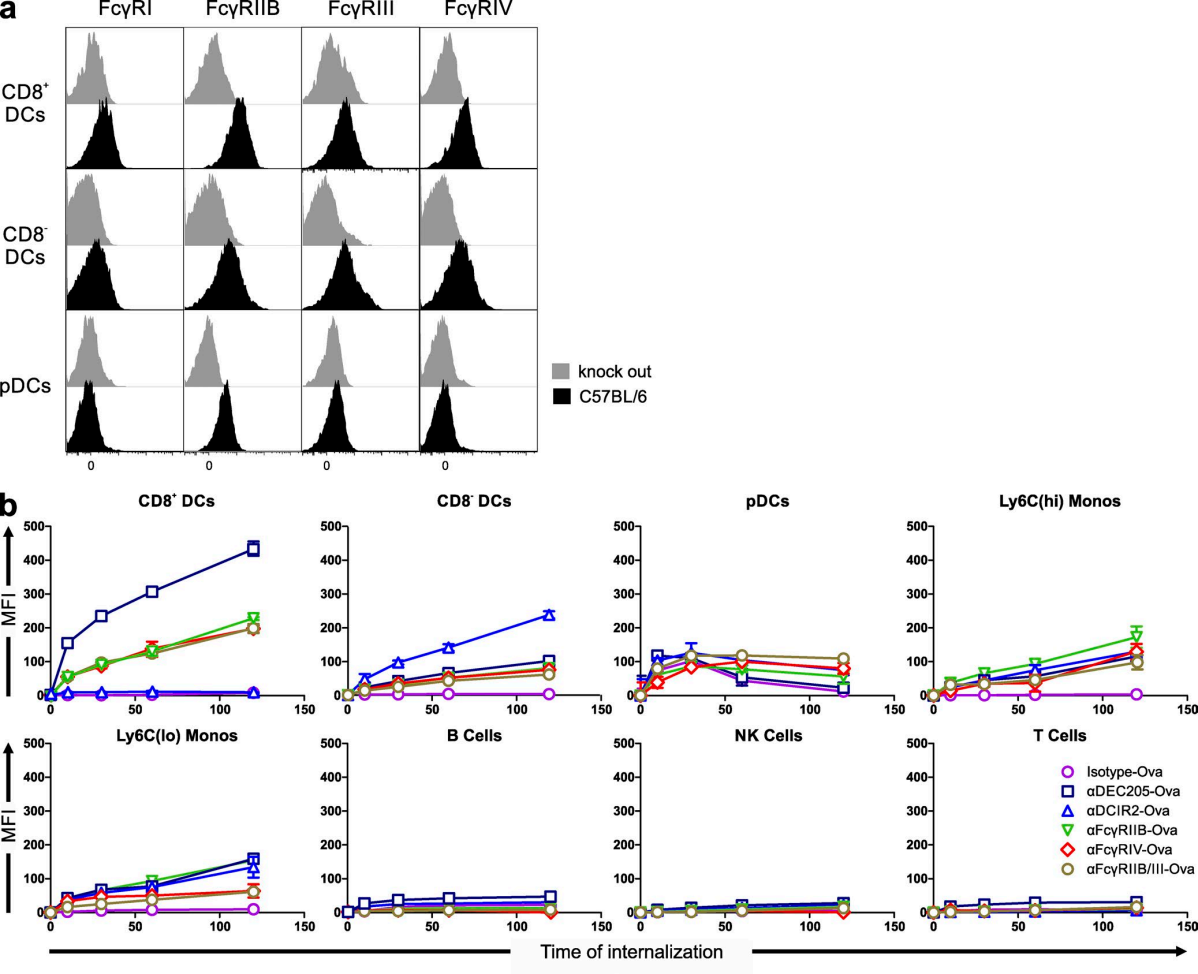
**Expression and internalization analyses of FcγRs.** (a) Histogram overlays show expression of FcγRs (FcγRI: CD64, FcγRIIB: CD32, FcγRIII: CD16, and FcγRIV: 9E9) on splenic single lin^−^MHCII^+^ CD11c^+^CD8^+^ DCs, CD11c^+^CD8^−^ DCs, and pDCs (see gating strategy in Fig. S1 a) from C57BL/6 (black) and FcγRI^−/−^, FcγRIIB^−/−^, FcγRIII^−/−^, and FcγRIV^−/−^ mice (gray). This experiment was repeated three times with similar results. (b) Internalization of αDEC205-Ova, αDCIR2-Ova, αFcγRIIB-Ova, αFcγRIV-Ova, αFcγRIIB/III-Ova, or isotype-Ova into the indicated splenic cell populations. Splenic single-cell suspensions were incubated in media containing the antibodies labeled with an oligo containing an Atto647N fluorochrome for 10, 30, 60, or 120 min at 37°C or kept on ice (0-min time point). Extracellular fluorescence was quenched by incubation with a complementary oligo containing a specific BBQ650 quencher. Cells were gated on single CD19^+^ B cells, CD11c^+^CD8^+^ DCs, CD11c^+^CD8^−^ DCs, CD11c^low^PDCA-1^+^B220^+^ pDCs, Ly6C^high^ inflammatory and Ly6C^low^ resident monocytes, T cells, and NK cells as described in Fig. S1 b. The presented experiments were repeated three times with two samples each. All data points ± SD are shown in the graph. MFI, mean fluorescence intensity.

Several groups have investigated the expression of FcγRs on mouse DCs with partially controversial findings. Therefore, we first examined FcγR expression on the three main splenic DC subpopulations in wild-type mice and their respective FcγR knockout mice. As shown in Fig. 1 a, all four FcγRs were expressed on CD11c^+^CD8^−^ and CD11c^+^CD8^+^ splenic DCs. In contrast, splenic pDCs solely expressed FcγRIIB, consistent with previous studies (Fig. 1 a and Fig. S1 a; Desai et al., 2007; Flores et al., 2009).

To be able to specifically study antigen uptake and presentation via select FcγRs, we chose FcγRIIB and FcγRIV as model receptors, as well-characterized monoclonal antibodies specific for these receptors and knockout mouse strains are available. Moreover, one of these receptors (FcγRIV) is an activating receptor, whereas the other (FcγRIIB) is the inhibitory FcγR. This allows for the study of whether Fc receptor signaling has an impact on the ensuing T cell response. In addition, we used an antibody specific for FcγRIIB/FcγRIII to target one inhibitory receptor and one activating Fc receptor in parallel. For the genetic fusion of the model antigen ovalbumin (Ova) with FcγR-specific antibodies, we cloned the variable regions of the αFcγRIIB, αFcγRIIB/III, and αFcγRIV antibodies. We further changed the constant part of the heavy chains (HCs) to a modified mouse IgG1 fragment incapable of being bound by FcγRs to prevent an antibody Fc-dependent interaction with other Fc receptors and genetically fused these antibodies with Ova, as we have done previously for the DC-specific targeting antibodies αDEC205 and αDCIR2 (Dudziak et al., 2007).

To study the endocytosis of the targeting antibodies (αDEC205-Ova, αDCIR2-Ova, αFcγRIIB-Ova, αFcγRIV-Ova, αFcγRIIB/III-Ova, or isotype-Ova) into various splenic cell populations, we labeled the antibodies with a specific oligo harboring an Atto647N dye as previously reported (Liu and Johnston, 2013; Reuter et al., 2015). After incubation of whole splenic single-cell suspensions for various periods of time, we quenched the outside fluorescence signal by adding a reverse complementary oligo harboring a BBQ650 quencher. Therefore, this method solely allows for the measurement of internalized antibodies. Overall, we found that CD11c^+^CD8^+^ DCs were more efficient in the uptake of FcγR-targeting antibodies than CD11c^+^CD8^−^ DCs. In addition, we found no differences in the uptake of FcγR antibodies when compared with each other. αDEC205- and αDCIR2-targeting antibodies were internalized as expected from previous results (Dudziak et al., 2007). The targeting antibodies were also taken up by Ly6C^high^ and Ly6C^low^ monocyte subpopulations as well as into pDCs. Further, we found no internalization into NK, B, or T cells of any of the targeting antibodies. B cells express high amounts of a nonendocytic variant of FcγRIIB, thus explaining the lack of uptake of FcγRIIB and FcγRIIB/III targeting antibodies (Fig. 1 b; Amigorena et al., 1992a).

### Antigen uptake via FcγRs induces T cell proliferation in vivo

**Figure 2.**
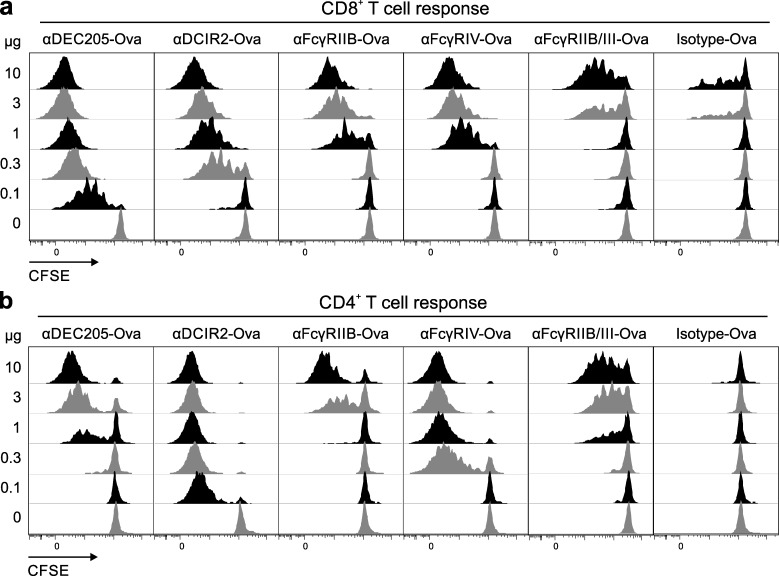
**In vivo proliferation of CD4^+^ and CD8^+^ T cells by antigen delivery to FcγRs in the steady state.** (a and b) MACS-purified, CFSE-labeled, congenic 10^6^ CD8^+^ OT-I T cells (a) or 2 × 10^6^ CD4^+^ OT-II T cells (b) were i.v. transferred into C57BL/6 mice. 16 h later, recipients were i.p. injected with doses of 10, 3, 1, 0.3, 0.1, and 0 µg isotype-Ova, αDEC205-Ova, αDCIR2-Ova, αFcγRIIB-Ova, αFcγRIV-Ova, or αFcγRIIB/III-Ova targeting antibodies in PBS. 3 d after targeting antibody injection, in vivo T cell proliferation in dependency of the injected antibody amount was measured by CFSE-dilution analysis via flow cytometry of Vα2^+^CD45.1^+^CD8^+^ (a) and Vα2^+^CD45.1^+^CD4^+^ (b) splenic T cells. All experiments were repeated at least three times with similar results.

Having demonstrated that the FcγRs IIB, III, and IV are able to internalize antigen–antibody conjugates (Fig. 1 b), we next assessed the capacity of these receptors to deliver the associated antigens into MHC class I and MHC class II antigen presentation pathways ultimately leading to the activation and proliferation of CD8^+^ or CD4^+^ T cells. Therefore, we transferred congenic antigen-specific CFSE-labeled CD4^+^ or CD8^+^ T cells into C57BL/6 mice followed by injection of different amounts of endotoxin-free αFcγRIIB-Ova, αFcγRIV-Ova, αFcγRIIB/III-Ova, or isotype-Ova antibodies 16 h later (Fig. 2). αDEC205-Ova and αDCIR2-Ova served as positive controls for CD8^+^ and CD4^+^ T cell proliferation, respectively (Dudziak et al., 2007). Three days after antibody injection, T cell proliferation was examined by analysis of CFSE dilution (Fig. 2). Although antigen delivery to the FcγRs resulted in dose-dependent CD8^+^ (Fig. 2 a) and CD4^+^ T cell (Fig. 2 b) proliferation three days after antigen delivery, FcγRIV-dependent CD4^+^ T cell responses were superior to those initiated via targeting of FcγRIIB or FcγRIIB/III. Thus, a 30-fold higher dose of αFcγRIIB-Ova as well as αFcγRIIB/III-Ova was necessary to induce a comparable CD4^+^ T cell response to αFcγRIV-Ova. Interestingly, αFcγRIV-Ova induced a CD4^+^ T cell expansion that was only slightly less efficient than that observed for αDCIR2-Ova and more efficient than the one induced by αDEC205-Ova (Fig. 2 b). In contrast, DEC205 targeting was most efficient for the induction of CD8^+^ T cell proliferation, followed by DCIR2, FcγRIV, FcγRIIB, and FcγRIIB/III (Fig. 2 a).

**Figure 3.**
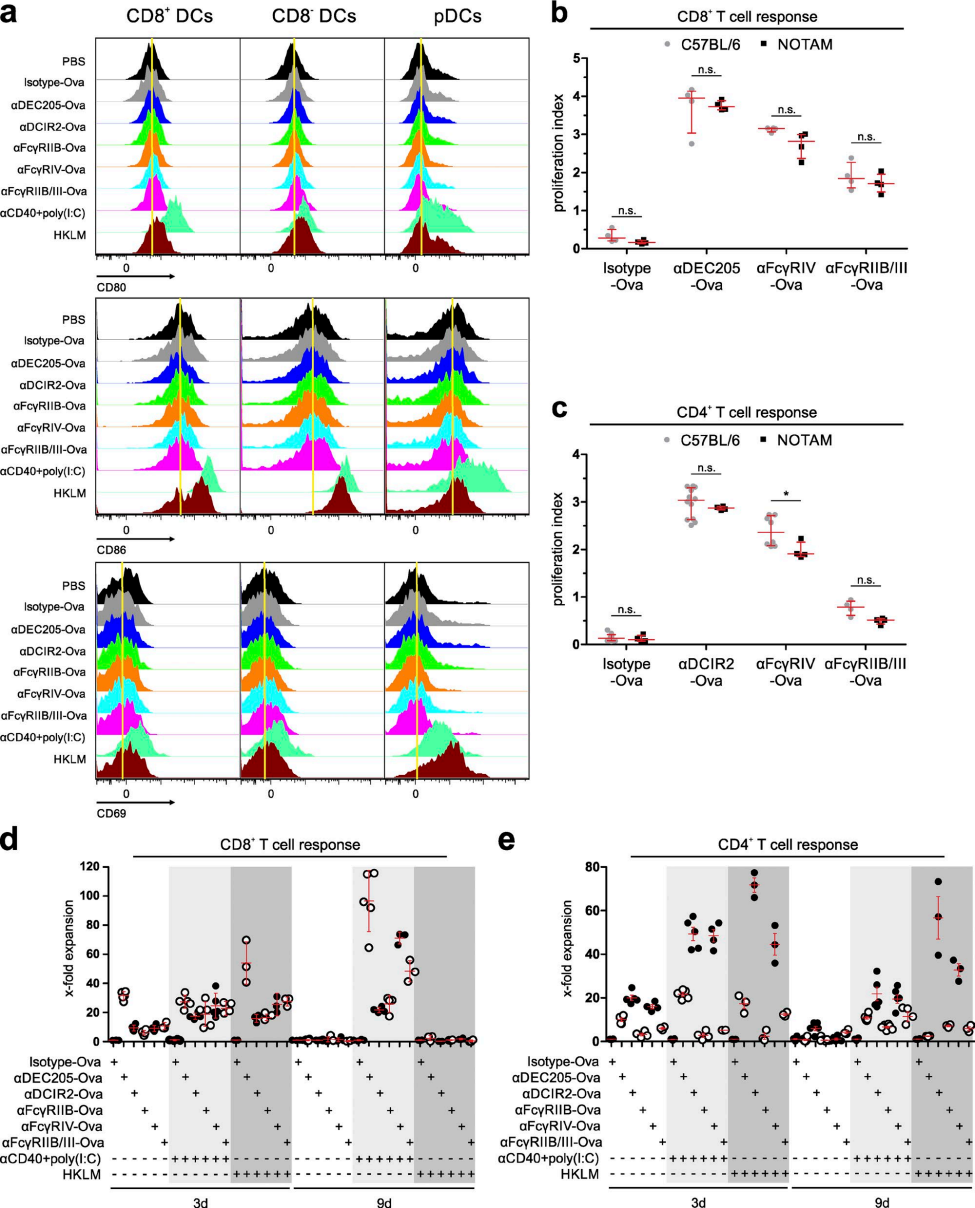
**T cell proliferation induced by FcγR targeting is ITAM independent, and long-term proliferative responses require additional co-stimulatory signals in vivo.** (a) Injection of antigen-targeting antibodies does not change activation status of DCs in vivo. 10 µg αDEC205-Ova, αDCIR2-Ova, αFcγRIIB-Ova, αFcγRIV-Ova, αFcγRIIB/III-Ova, or isotype-Ova, was i.p. injected into C57BL/6 mice. As positive control, a mixture of each 25 µg poly(I:C) and αCD40 or 10^9^ HKLM was used. PBS was i.p. injected as negative control. 12 h later, splenic single lin^−^ (CD3^−^CD19^−^NKp46^−^) MHCII^+^ CD11c^+^CD8^+^ DCs, CD11c^+^CD8^−^ DCs, and CD11c^low^PDCA1^+^ pDCs were analyzed by flow cytometry for the activation markers CD80, CD86, and CD69. Data were analyzed using DIVA and FlowJo Software. This experiment was repeated more than five times with similar results and was used as quality control in the production of antigen-targeting antibodies. (b–e) MACS-purified, CFSE-labeled, congenic 10^6^ CD8^+^ OT-I T cells (b and d) or 2 × 10^6^ CD4^+^ OT-II T cells (c and e) were i.v. transferred into C57BL/6 mice (b–e) or NOTAM mice (b and c). 16 h later, recipients were i.p. injected with 3 µg of the targeting antibodies in PBS (b and c) or together with 25 µg αCD40 antibody and 12.5 µg poly(I:C) (pIC) or HKLM (d and e). (b and c) 3 d after targeting antibody injection, in vivo T cell proliferation was measured by CFSE-dilution analysis via flow cytometry of Vα2^+^CD45.1^+^CD8^+^ (b) and Vα2^+^CD45.1^+^CD4^+^ (c) splenic T cells. The graph shows the proliferation indices of all mice analyzed (n.s., nonsignificant; *, P < 0.05). (d and e) T cell proliferation was analyzed by cell numbers of gated Vα2^+^CD45.1^+^CD8^+^ (d) or Vα2^+^CD45.1^+^CD4^+^ (e) splenic T cells 3 d and 9 d later, when PBS, αCD40 + poly(I:C), or HKLM was coinjected. Graphs show the relative cell number expansion compared with the isotype control. (a) These experiments were repeated at least three times with similar results. (b–e) The data were generated within three independent experiments, and all data points ± SD are presented (n.s., nonsignificant; *, P < 0.05; Mann–Whitney *U* test).

To study, whether the observed differences in the T cell responses were caused by differential induction of signaling events within the DCs, we first analyzed changes in the DC maturation status induced by the targeting antibodies. To examine this, we injected the targeting antibodies into naive mice and analyzed the expression of CD40, CD69, and CD80 on CD11c^+^CD8^+^ and CD11c^+^CD8^−^ DCs as well as on pDCs (Dudziak et al., 2007; Gao et al., 2009; Alari-Pahissa et al., 2012). As shown in Fig. 3 a, none of the antibodies affected the maturation status of the three splenic DC subpopulations. To further rule out the influence of possible ITAM-dependent signaling effects on the T cell responses induced by targeting of activating FcγRs, we took advantage of a mouse, in which the endogenous FcRγ-chain is replaced by a variant with a nonfunctional ITAM. This results in normal FcγR cell surface expression but uncouples the transduction of ITAM-dependent signaling pathways from antibody binding to activating FcγRs (de Haij et al., 2010; Boross et al., 2014). As shown in Fig. 3 b, the transfer of CFSE-labeled transgenic CD4^+^ or CD8^+^ T cells into C57BL/6 or NOTAM mice followed by injection of the targeting antibodies (αFcγRIV-Ova, αFcγRIIB/III-Ova, αDEC205-Ova, αDCIR2-Ova, or isotype-Ova) resulted in either no or only slight differences in the induced T cell responses in NOTAM mice compared with wild-type C57BL/6 mice. This suggests that ITAM signaling has no major influence on the T cell responses induced by antigen-targeting antibodies specific for activating FcγRs (Fig. 3, c and d).

**Figure 4.**
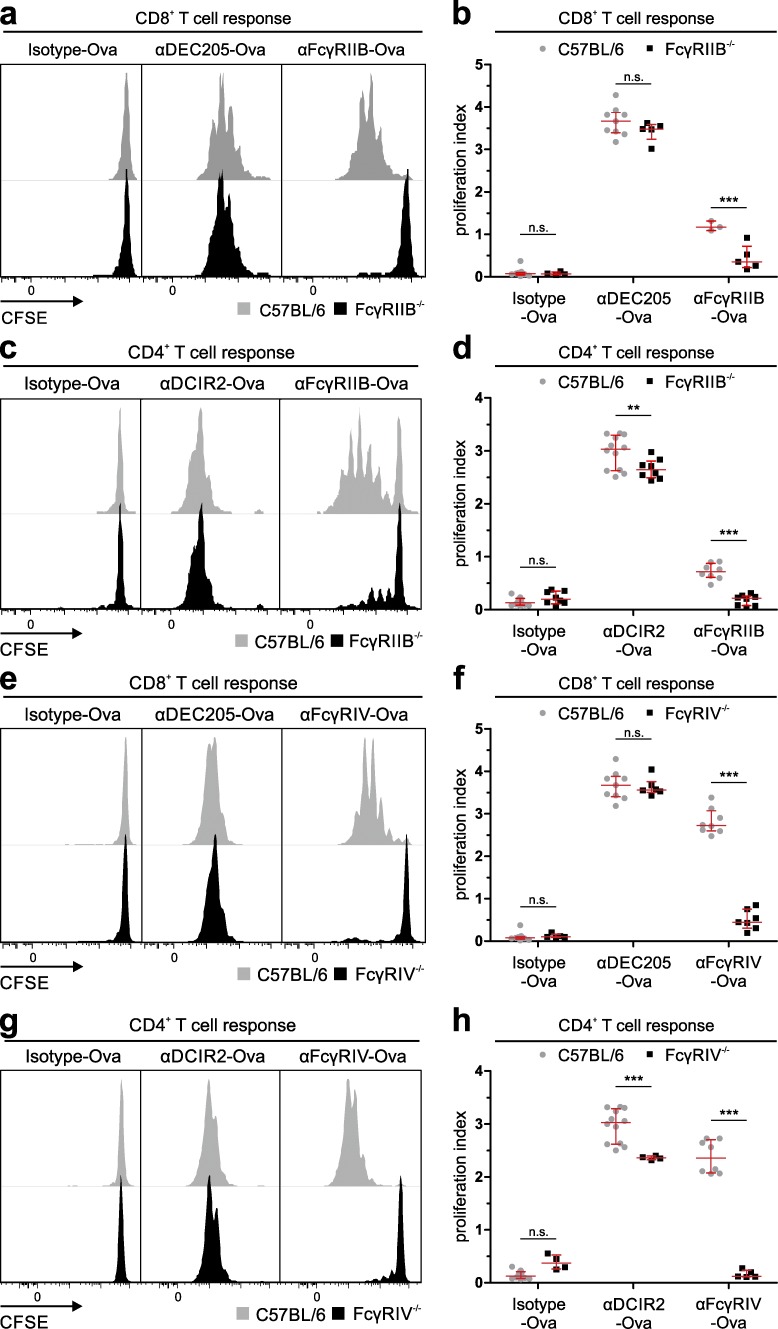
**Induction of T cell responses by FcγR targeting is dependent on the presence of FcγRs.** (a–h) C57BL/6, FcγRIIB^−/−^ (a–d), or FcγRIV^−/−^ (e–h) mice were i.v. injected with congenic10^6^ CD45.1^+^CD8^+^ OT-I (a, b, e, and f) or 2 × 10^6^ CD45.1^+^CD4^+^ OT-II (c, d, g, and h) MACS-purified, CFSE-labeled T cells. 3 µg of the antigen-targeting antibodies αDEC205-Ova, αDCIR2-Ova, and αFcγRIV-Ova or 10 µg αFcγRIIB-Ova or isotype-Ova was i.p. injected 16 h after T cell transfer. T cell proliferation was analyzed by CFSE dilution in gated Vα2^+^CD45.1^+^CD8^+^ OT-I (a, b, e, and f) or Vα2^+^CD45.1^+^CD4^+^ OT-II (c, d, g, and h) T cells 72 h later. (a, c, e, and g) Shown are exemplary overlay histograms (gray: C57BL/6, black: [a and c] FcγRIIB^−/−^, [e and g] FcγRIV^−/−^) of at least three independent experiments with similar results. (b, d, f, and h) Scatter plots represent the proliferation indices of all mice analyzed ± SD (gray circles: C57BL/6, black squares: [b and d] FcγRIIB^−/−^, [f and h] FcγRIV^−/−^). (n.s., nonsignificant; **, P < 0.01; ***, P < 0.001 by Mann–Whitney *U* test.)

**Figure 5.**
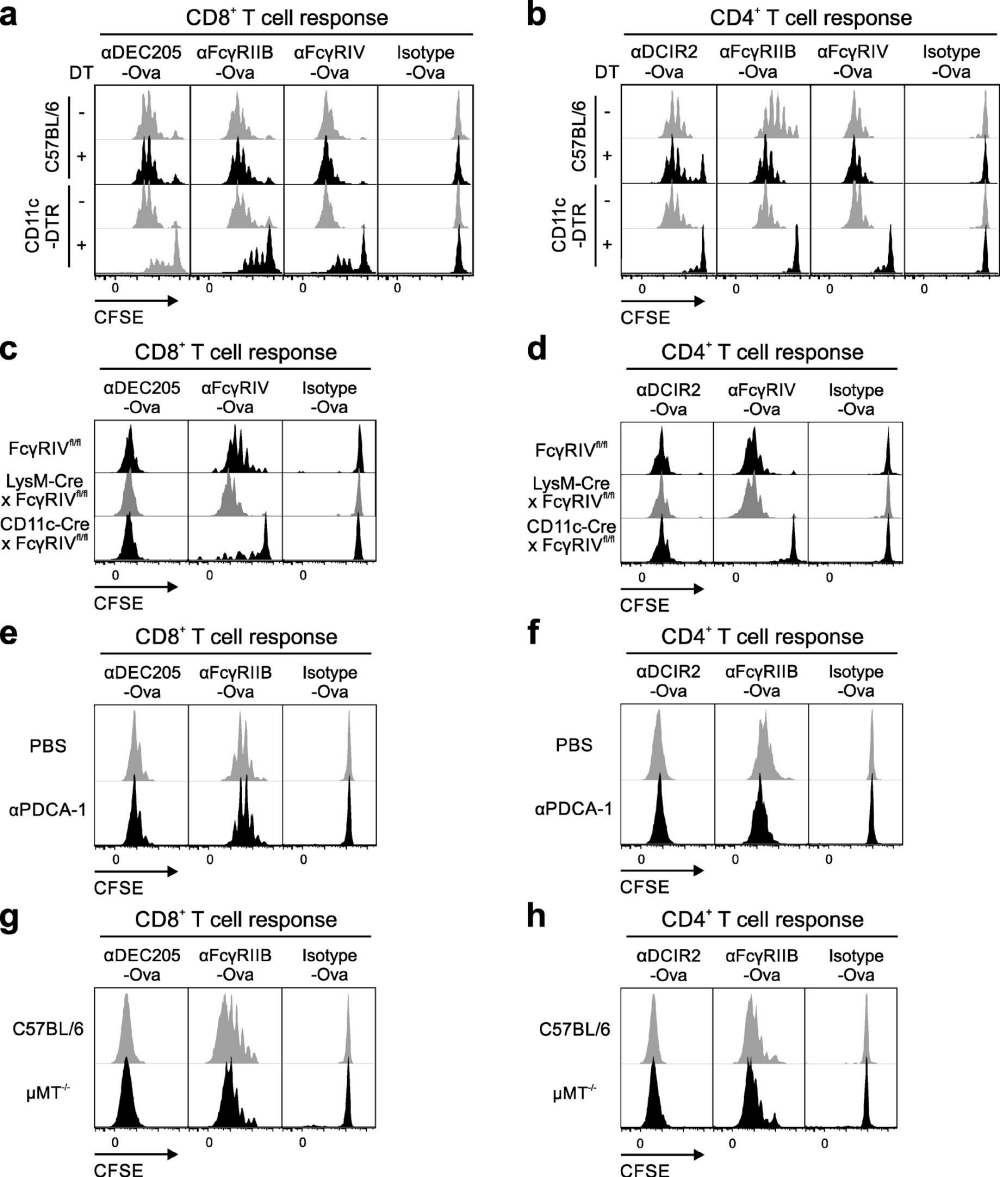
**Antigen targeting to FcγRIIB and FcγRIV needs receptor expression on cDCs**. (a–h) Mice were i.v. injected with MACS-purified, CFSE-labeled 10^6^ CD45.1^+^CD8^+^ OT-I (a, c, e, and g) or 2 × 10^6^ CD45.1^+^CD4^+^ OT-II (b, d, f, and h) T cells. 3 µg αDEC205-Ova, αDCIR2-Ova, or αFcγRIV-Ova or 10 µg αFcγRIIB-Ova or isotype-Ova control antibodies was i.p. injected 16 h after T cell transfer. T cell proliferation was evaluated 72 h later. Shown is the CFSE dilution of Vα2^+^CD45.1^+^CD8^+^ OT-I (a, c, e, and g) or Vα2^+^CD45.1^+^CD4^+^ OT-II (b, d, f, and h) gated congenic T cells. (a and b) C57BL/6 mice and CD11c-DTR mice were treated i.p. with PBS or DT 8 h before T cell transfer. (c and d) T cell transfer into FcγRIV^fl/fl^, LysM-Cre × FcγRIV^fl/fl^, CD11c-Cre × FcγRIV^fl/fl^ mice. (e and f) C57BL/6 mice were treated three times with 200 µg αPDCA-1 antibody 64 h, 40 h, and 16 h before T cell transfer. (g and h) T cell transfer into C57BL/6 or µMT^−/−^ mice. All experiments were repeated at least three times with similar results.

**Figure 6.**
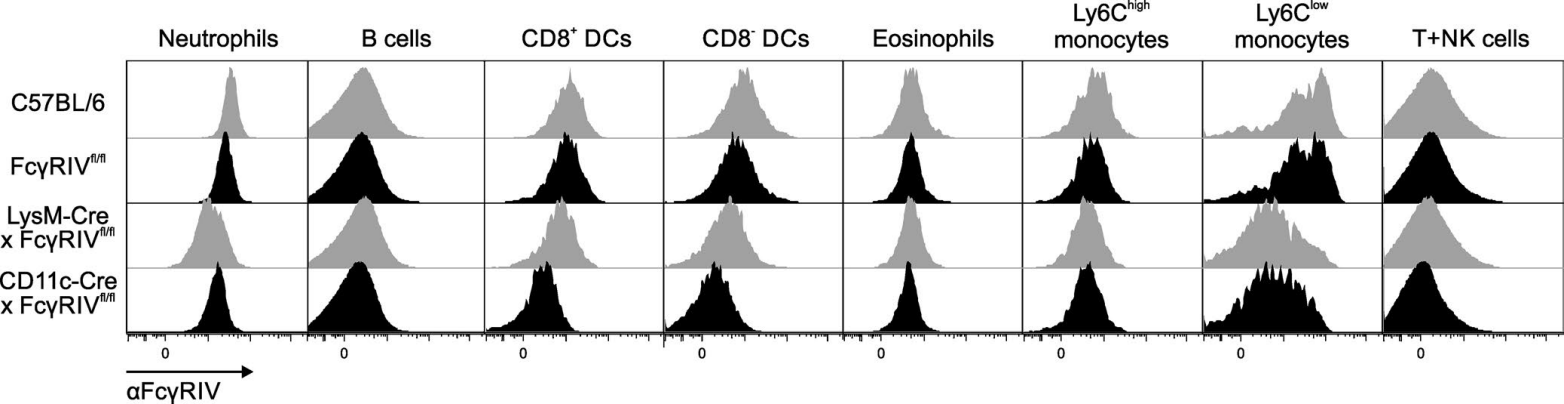
**CD11c-Cre × FcγRIV^fl/fl^ and LysM-Cre × FcγRIV^fl/fl^ deplete FcγRIV on different cell types.** Freshly isolated splenic single-cell suspensions from C57BL/6, FcγRIV^fl/fl^, CD11c-Cre × FcγRIV^fl/fl^, and LysM-Cre × FcγRIV^fl/fl^ were stained with antibodies for cell identification and for FcγRIV similar as shown in Fig. S1. Ly6G was used in an additional channel, and neutrophils were gated as Ly6G^hi^SSC^im/hi^ and excluded before starting to gate the other populations. The experiments were repeated five times with similar results.

**Figure 7.**
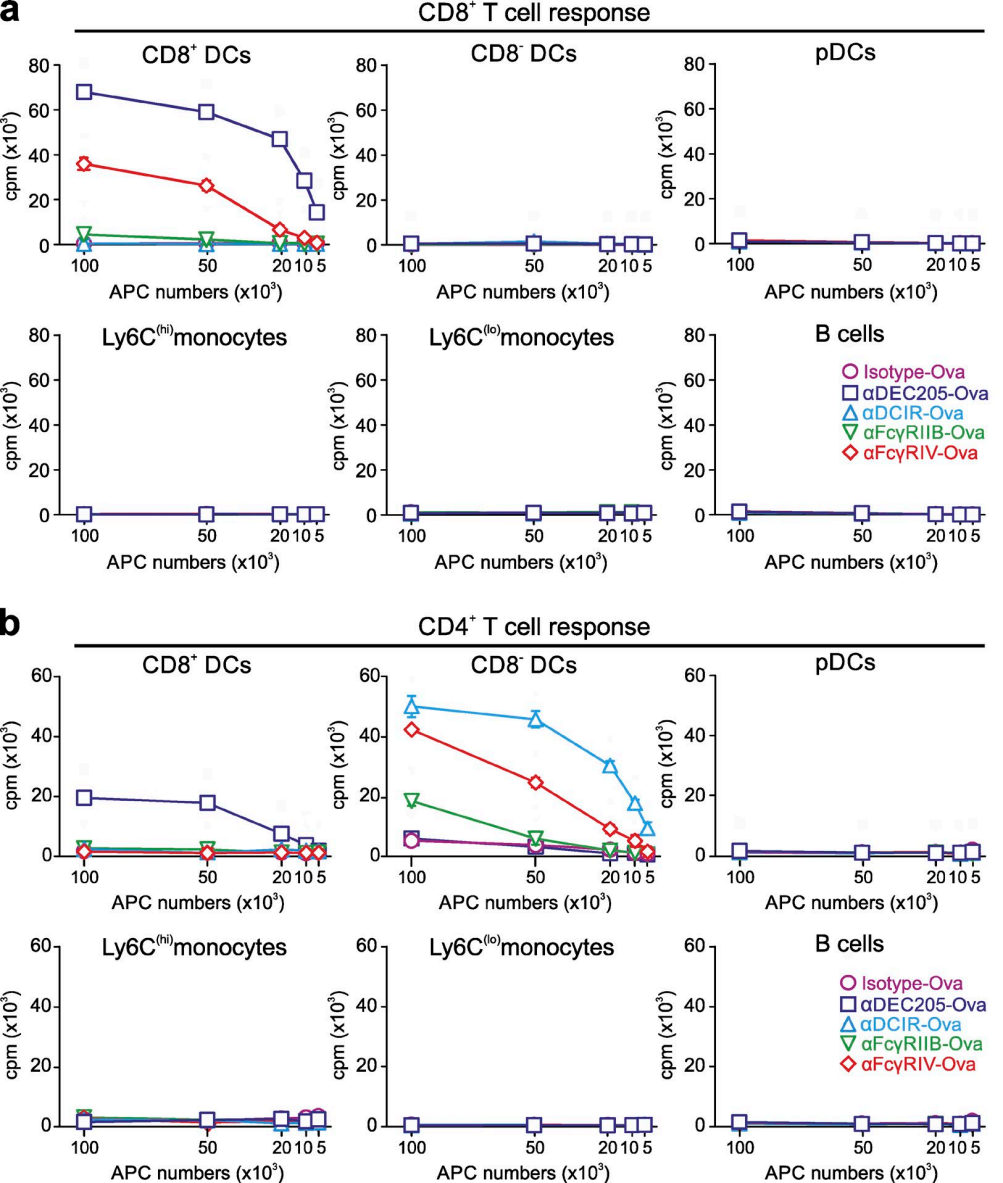
**Differential antigen presentation to CD8^+^ and CD4^+^ T cells induced by FcγRIIB and FcγRIV targeting to CD11c**^+^**CD8**^+^ **or CD11c**^+^**CD8^−^ DCs.** 10 µg αDEC205-Ova, αDCIR2-Ova, or αFcγRIV-Ova or 30 µg αFcγRIIB-Ova or isotype-Ova was i.p. injected into C57BL/6 mice. 12 h later, splenocytes were sorted into CD11c^+^CD8^+^ DCs, CD11c^+^CD8^−^ DCs, pDCs, B cells, and Ly6C^high^ and Ly6C^low^ monocytes. (a and b) Antigen-presenting cells were co-cultured in different numbers with 10^5^ MACS-enriched CD8^+^ OT-I T cells (a) or CD4^+^ OT-II T cells (b). Proliferation was evaluated by addition of ^3^H-thymidine 16 h (a) or 40 h (b) after start of the co-culture. Incorporation of ^3^H-thymidine was measured 24 h later. This experiment was repeated at least three times, and all data points ± SD are shown in the graph.

We have demonstrated before that this initial proliferation at day 3 does not directly translate into long-term proliferation and therefore antigen-specific immunity (Dudziak et al., 2007; Soares et al., 2007). In fact, when antigen is delivered to DCs without any co-stimulatory signal, T cells proliferate until day 3 and are then lost until day 9. To investigate, whether antigen uptake via the different FcγRs is able to induce long-term proliferative responses of antigen-specific T cells under stimulatory conditions in vivo*,* we transferred congenic antigen specific T cells into C57BL/6 mice and injected 3 µg of the targeting antibodies 8 h later in the presence or absence of a stimulatory αCD40 antibody in combination with the TLR3 ligand polyinosinic:polycytidylic acid (poly(I:C); αCD40/pIC) as described before (Fig. 3, d and e; Boscardin et al., 2006) or 10^9^ heat-killed *Listeria monocytogenes* (HKLM). Interestingly, 3 d after priming, the αDEC205-Ova targeting was superior in the induction of CD8^+^ T cell responses in the absence of adjuvant, whereas all CD8^+^ T cell responses were comparable when αCD40/pIC was injected additionally (Fig. 3 d). Notably, αDCIR-Ova and αFcγRIV-Ova targeting induced efficient CD4^+^ T cell responses, which were three times stronger when the antibodies were injected together with αCD40/pIC (Fig. 3 e). Nine days after T cell transfer, CD4^+^ and CD8^+^ antigen-specific T cell numbers were strongly reduced, when the antibodies were injected without co-stimulatory αCD40/pIC (Fig. 3, d and e). In contrast, when the priming was performed in the presence of αCD40/pIC, antigen-specific T cells persisted, and especially FcγRIV-dependent CD4^+^ and CD8^+^ T cell responses were dramatically enhanced and became comparable to those observed with αDEC205-Ova or αDCIR2-Ova antibodies, respectively. This suggests that antigens taken up via FcγRIV are efficiently directed into MHC class I and MHC class II antigen presentation pathways (Fig. 3, d and e; Ukkonen et al., 1986; Amigorena et al., 1992a,b). In contrast, targeting antigens to FcγRIIB as well as to FcγRIIB/III was less efficient in inducing CD4^+^ and CD8^+^ T cell responses (Figs. 2 and 3, d and e). Nonetheless, for FcγRIIB, a CD8^+^ T cell response comparable to that induced by antigen delivery via αDCIR2-Ova could be detected, whereas FcγRIIB/III targeting induced a stronger CD8^+^ T cell proliferation compared with DCIR2 targeting. Moreover, testing HKLM as a more natural adjuvant revealed its potency as co-stimulator for CD4^+^ T cell proliferation, as it was even better than αCD40/pIC for αDCIR2-Ova and αFcγRIV-Ova targeting. However, HKLM co-stimulation was not efficient for inducing long-term CD8^+^ T cell proliferation (Fig. 3, d and e). As the targeting antibodies αFcγRIIB-Ova and αFcγRIV-Ova bind only a single inhibitory or activating FcγR, we focused on these receptors for more in-depth mechanistic analyses (Figs. 4, 5, 6, and 7).

Confirming that antigen delivery via FcγRIIB- and FcγRIV-specific antibodies was indeed only occurring by active uptake via the respective FcγRs and not through a receptor-independent phagocytosis or endocytosis process, no T cell proliferation could be observed in FcγRIIB- and FcγRIV-deficient animals after injection of the respective αFcγR antibodies. As expected, a normal T cell response was observed when αDEC205-Ova or αDCIR2-Ova antibodies were used in FcγRIIB^−/−^ or FcγRIV^−/−^ mice (Fig. 4, a–h).

### Antigen presentation by DCs is responsible for the initiation of T cell responses

As neither FcγRIIB nor FcγRIV expression is restricted to DCs (Nimmerjahn et al., 2005; Biburger et al., 2011) and as we could show that other innate immune effector cells, including monocytes, take up the antigen–antibody construct ex vivo (Fig. 1 b), we next assessed which cell types contributed to the induction of T cell proliferation. To study the role of DCs in this process, we used CD11c-DTR mice, in which DCs can be deleted by injection of diphtheria toxin (DT; Hochweller et al., 2008). First, we analyzed the efficacy of DT in depleting DCs in these mice and demonstrated that CD11c^+^CD8^+^ and CD11c^+^CD8^−^ DCs were efficiently depleted (Fig. S2 a). We also studied the DC maturation state and found no up-regulation of co-stimulatory molecules by DT injection (Fig. S2 b and not depicted). Next, congenic antigen-specific CD8^+^ (Fig. 5 a) or CD4^+^ CFSE-labeled T cells (Fig. 5 b) were transferred into DT- or PBS-treated CD11c-DTR and C57BL/6 mice followed by injection of αDEC205-Ova, αDCIR2-Ova, αFcγRIIB-Ova, αFcγRIV-Ova, or isotype-Ova antibodies 8 h later. As shown in Fig. 5 (a and b), we found a marked reduction of CD8^+^ and a virtually complete abrogation of CD4^+^ T cell proliferation in DC-depleted CD11c-DTR, but not wild-type control or PBS-treated CD11c-DTR animals in vivo (Fig. 5, a and b).

More recently, FcγRIV-floxed mice became available, enabling a cell type–specific deletion of FcγRIV (Seeling et al., 2013). To generate mice with a macrophage- or DC-specific deletion of FcγRIV, we generated LysM-Cre × FcγRIV^fl/fl^ and CD11c-Cre × FcγRIV^fl/fl^ animals. As shown in Fig. 6, LysM-Cre × FcγRIV^fl/fl^ mice lost FcγRIV expression on Ly6C^low^ monocytes and showed a reduced expression on neutrophils, CD11c^+^CD8^+^ DCs, and CD11c^+^CD8^−^ DCs (Fig. 6). In contrast, CD11c-Cre × FcγRIV^fl/fl^ mice are negative for FcγRIV expression on Ly6C^low^ monocytes and both cDC subsets (Fig. 6). Of note, only CD11c-Cre × FcγRIV^fl/fl^ mice showed an abrogation of CD4^+^ and CD8^+^ T cell proliferation upon antigen delivery to FcγRIV (Fig. 5, c and d), whereas T cell responses were comparable in wild-type and LysM-Cre × FcγRIV^fl/fl^ mice. As before, the induction of CD4^+^ and CD8^+^ T cell responses was normal in animals with a cell type–specific deletion of FcγRIV, when αDEC205-Ova or αDCIR2-Ova antibodies were used to deliver the antigen to DCs (Fig. 5, c and d).

With respect to FcγRIIB, not only cDCs but also pDCs show a prominent expression of this receptor and thus may be responsible for the induction of T cell responses (Loschko et al., 2011). To test this, we depleted pDCs via injection of an αPDCA-1 antibody before T cell transfer (Fig. 5, e and f; Fig. S2 c; and not depicted). Although the pDC population was reduced by 90% (Fig. S2 c), we found no difference in the CD8^+^ and CD4^+^ antigen-specific T cell proliferation in αPDCA-1 antibody–treated animals in comparison to PBS-treated controls (Fig. 5, e and f; and not depicted), suggesting that pDCs might not be critical for αFcγRIIB-Ova–mediated induction of T cell responses. Besides DCs, B cells express high levels of FcγRIIB and thus may contribute to T cell stimulation. However, as depicted in Fig. 5 (g and h), B cell–deficient µMT^−/−^ and C57BL/6 control mice showed indistinguishable T cell responses, therefore excluding that B cells are involved in the initiation of T cell proliferation in our system. This is consistent with the expression of the FcγRIIB1 isoform on B cells, which cannot internalize bound ligands as also shown in Fig. 1 b (Amigorena et al., 1992a).

### DC subpopulation–specific induction of CD8^+^ and CD4^+^ T cell responses by FcγRIV and FcγRIIB targeting

As CD11c^+^CD8^+^ and CD11c^+^CD8^−^ DC subpopulations express both FcγRIIB and FcγRIV, we determined whether both DC subsets were involved in the initiation of CD8^+^ and CD4^+^ T cell responses. To address this question, we sorted splenic CD11c^+^CD8^+^ DCs, CD11c^+^CD8^−^ DCs, pDCs, Ly6C^high^, Ly6C^low^ monocytes, and B cells 12 h after i.p. injection of mice with 10 µg αDEC205-Ova, αDCIR2-Ova, or αFcγRIV-Ova or 30 µg αFcγRIIB-Ova or isotype-Ova antibodies and cultured them in the presence of CD4^+^ and CD8^+^ antigen-specific T cells in vitro (Fig. 7, a and b). Confirming our previous results (Fig. 5), pDCs, monocytes, or B cells were not able to stimulate T cell proliferation. Quite interestingly, and despite expression of FcγRIV on both DC subsets, only the CD11c^+^CD8^+^ DC subset was able to stimulate FcγRIV-dependent CD8^+^ T cell responses, whereas the CD11c^+^CD8^−^ DC subset triggered CD4^+^ T cell proliferation (Fig. 7, a and b). This suggests that antigens endocytosed via FcγRIV on CD11c^+^CD8^+^ DCs are largely shuttled into the cross-presentation pathway, whereas FcγRIV-dependent endocytosis into CD11c^+^CD8^−^ DCs results in a dominant loading onto MHC class II molecules. Although rather low, a similar pattern of T cell stimulation was observed for antigens delivered to DCs via the inhibitory FcγRIIB (Fig. 7, a and b).

Overall, these data support a model in which the DC subsets may be specialized to preferentially induce either a CD8^+^ or CD4^+^ T cell response, respectively. This is consistent with our previous study showing that CD11c^+^CD8^+^ DCs were responsible for the CD8^+^ T cell response upon αDEC205-Ova targeting, whereas the CD4^+^ T cell response induced via αDCIR2-Ova was exclusively initiated via the CD11c^+^CD8^−^ DCs subset in the steady state (Dudziak et al., 2007; Soares et al., 2007).

### Induction of effector CD4^+^ and CD8^+^ T cell responses in naive animals after FcγR-mediated antigen delivery

**Figure 8.**
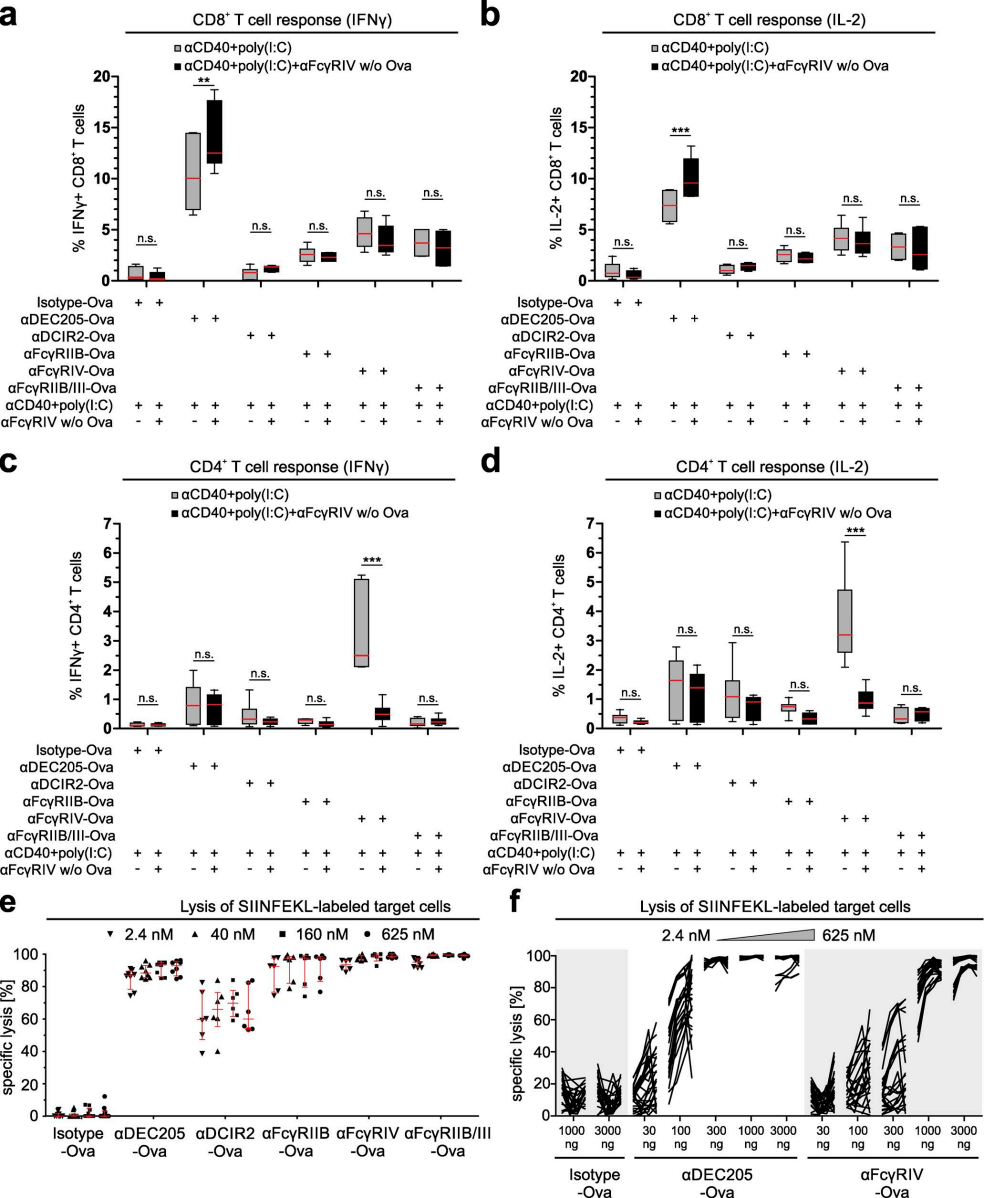
**Induction of effector T cell responses in naive mice mediated by antigen delivery through FcγRs.** (a–d) C57BL/6 mice were i.p. injected with 10 µg of the targeting antibodies αDEC205-Ova, αDCIR2-Ova, αFcγRIIB-Ova, αFcγRIV-Ova, αFcγRIIB/III-Ova, or isotype-Ova together with 50 µg αCD40 antibody and 25 µg poly(I:C) after injection of PBS or 200 µg αFcγRIV w/o Ova. 14 d later, splenocytes were in vitro restimulated for 12 h with freshly isolated CD11c-positive MACS-enriched DCs loaded with a peptide pool of Ova. Intracellular IFNγ (a and c) and IL-2 (b and d) production was analyzed by flow cytometry. The graphs represent the number of cytokine-positive TCRβ^+^NKp46^−^CD19^−^CD8^+^CD4^−^ (a and b) and TCRβ^+^NKp46^−^CD19^−^CD8^−^CD4^+^ (c and d) T cells. This experiment was performed twice with at least five mice, and all data points were used for the analysis. Shown is the median ± interquartile range (n.s., nonsignificant; **, P < 0.01; ***, P < 0.001; Mann–Whitney *U* test). (e and f) C57BL/6 mice were immunized with 3 µg αDEC205-Ova, αDCIR2-Ova, αFcγRIV-Ova, or αFcγRIIB/III-Ova or 10 µg αFcγRIIB or isotype-Ova in combination with 50 µg αCD40 and 25 µg poly(I:C) (e) or 0.03, 0.10, 0.3, 1, or 3 µg αDEC205-Ova, αDCIR2-Ova, αFcγRIV-Ova, or αFcγRIIB/III-Ova or 10 µg αFcγRIIB or isotype-Ova in combination with 50 µg αCD40 and 25 µg poly(I:C) (f). 8 d later, mice were challenged i.v. with a cocktail of freshly isolated CD45.1^+^ splenocytes labeled with different concentrations of CFSE and/or cell trace violet and loaded with 2.4, 40, 160, or 625 nM SIINFEKL peptide (unloaded cells were used as injection control and control for specificity of the lysis). This allowed for the simultaneous analysis of target cell lysis loaded with different amounts of SIINFEKL within one mouse. 16 h later, splenocytes were analyzed for the presence of the transferred CD45.1^+^ cells. The data points shown in this graph have been generated within three independent experiments. Each dot represents the degree of lysis observed for splenocytes loaded with a specific amount of peptide (median ± interquartile range in e and one line for each single mouse in f). (e) This experiment was performed three times with two mice per group, and all data points are shown in the graphs. (f) This experiment was performed three times with three to five mice per group, and all data points are shown in the graphs.

Finally, we wanted to evaluate the potential of FcγR-mediated antigen delivery in a naive system without transfer of antigen-specific T cells. For this, C57BL/6 animals were i.p. injected with αDEC205-Ova, αDCIR2-Ova, αFcγRIIB-Ova, αFcγRIIB/III-Ova, αFcγRIV-Ova, or isotype-Ova in the presence of αCD40/pIC. After 14 d, splenocytes were restimulated with sorted CD11c^+^ DCs loaded with an overlapping Ova peptide pool (Fig. 8, a–d). As determined by intracellular cytokine staining, targeting to the different FcγRs induced the differentiation of naive CD8^+^ T cells into CD8^+^ T cells producing IFNγ and IL-2 after restimulation, although at much lower levels compared with αDEC205-Ova–treated animals (Fig. 8, a and b). Interestingly, αFcγRIV-Ova targeting was superior to all other antigen-targeting antibodies in the differentiation of naive CD4^+^ T cells into CD4^+^ T cells producing IFNγ and IL-2 after restimulation (Fig. 8, c and d), firmly establishing the important role of FcγRIV for the initiation of antigen-specific CD4^+^ T cell responses in vivo.

As FcγRIV targeting was superior for the induction of naive CD4^+^ T cell responses (especially compared with DCIR2 targeting), we were interested in FcγRIV as a potential co-stimulator for other targeting antibodies. We therefore injected all Ova-containing antibodies with 20-fold excess of an FcγRIV targeting antibody without Ova (αFcγRIV w/o Ova). However, this did not result in increased T cell responses upon targeting of FcγRIIB, FcγRIIB/III, and DCIR2. As expected, the additional injection of αFcγRIV w/o Ova blocked the response by FcγRIV-Ova targeting. In contrast, we observed a slight increase of the CD8^+^, but not the CD4^+^ T cell response induced by αDEC205-Ova targeting when we coinjected the αFcγRIV w/o Ova antibody, which will be subject to further investigations (Fig. 8, a–d).

To directly prove that the induced CD8^+^ T cell responses resulted in the development of fully functional effector T cells, we performed an in vivo killing assay (Fig. 8, e and f). For this assay, naive C57BL/6 animals were injected with the targeting antibodies αDEC205-Ova, αDCIR2-Ova, αFcγRIIB-Ova, αFcγRIIB/III-Ova, αFcγRIV-Ova, or isotype-Ova in the presence of αCD40/pIC. On day 8 after immunization, we transferred a mixture of fluorescently labeled, freshly isolated CD45.1^+^ splenocytes loaded with five different concentrations of the SIINFEKL peptide (0–625 nM) into the very same animals. 16 h later, the efficacy of antigen-specific target cell lysis was measured by flow cytometry. We found efficient target cell lysis after immunization with αDEC205-Ova, αFcγRIIB/III-Ova, and αFcγRIV-Ova, whereas targeting by αFcγRIIB-Ova was slightly less and αDCIR2-Ova least efficient (Fig. 8 e). Furthermore, we performed in vivo killing experiments with for αDEC205-Ova and αFcγRIV-Ova and doses of 30, 100, 300, 1,000, and 3,000 ng to elucidate dose dependency of induced CD8^+^ T cell responses. Our data revealed that three times more αFcγRIV-Ova than αDEC205-Ova was needed to induce a similar efficient cytotoxic response (Fig. 8 f), which in accordance to response of transgenic (Fig. 2 a) and naive CD8^+^ T cells (Fig. 8, a and b). Collectively, targeting of FcγRIV is able to induce a multimodal immune response.

## Discussion

Targeting antigens to DCs in vivo provides an efficient means to induce CD4^+^ and CD8^+^ T cell responses (Heidkamp et al., 2016a; Lehmann et al., 2016). Besides delivery of antigens to C-type lectin receptors, several studies have suggested that engaging Fc receptors on DCs ex vivo in the form of an immune complex may result in a superior priming of T cell responses in comparison to free antigen. Moreover, DCs deficient in the inhibitory FcγRIIB were able to induce enhanced T cell responses, supporting the notion that activating and inhibitory FcγRs set a threshold for DC activation, thereby modulating the strength of the resulting T cell response to antigens within immune complexes (den Haan and Bevan, 2002; Kalergis and Ravetch, 2002; Steinman et al., 2003a; Boruchov et al., 2005; Dhodapkar et al., 2005; Nimmerjahn and Ravetch, 2008). However, not much is known about the expression of activating and inhibitory FcγRs on splenic DC subsets and about the capacity of individual Fc receptors expressed on DCs to deliver antigens into MHC class I and MHC class II presentation pathways to stimulate CD4^+^ and CD8^+^ T cell responses. To be able to understand the specific properties of individual activating and inhibitory Fc receptors, we made use of well-characterized antibodies specific for FcγRIIB (inhibitory), FcγRIV (activating), and FcγRIIB/III (inhibitory and activating).

With respect to expression, the majority of studies only assessed FcγRI, FcγRIIB, and FcγRIII on CD11c^+^ DCs and some select DC subsets or receptors (Steinman and Cohn, 1974; Steinman et al., 1979; Ruedl et al., 1996; Regnault et al., 1999; den Haan and Bevan, 2002; Tan et al., 2003; de Jong et al., 2006; Desai et al., 2007; Björck et al., 2008; Hartwig et al., 2010; Langlet et al., 2012; Plantinga et al., 2013), whereas there is only limited knowledge of the expression of FcγRIV. This activating FcγR was identified only recently and, based on amino acid sequence and functional properties, is most closely related to human FcγRIIIA (Nimmerjahn et al., 2005; Syed et al., 2009). Thus, we first defined the pattern of FcγR expression on different splenic DC subsets by comparing wild-type and respective FcγR-deficient mouse strains, showing that all four FcγRs (FcγRI–IV) were present on cDC subpopulations (Fig. 1 a). The presence of FcγRIV, which plays an essential role for IgG2a and IgG2b immune complex–dependent proinflammatory effector functions, may allow for a feedback loop to DCs, thus modifying the resulting T cell responses (Boruchov et al., 2005; Nimmerjahn et al., 2005, 2010; Desai et al., 2007; Flores et al., 2009; Syed et al., 2009; Hartwig et al., 2010; Wilson et al., 2011). On pDCs, the expression of FcγRI, FcγRIII, and FcγRIV was undetectable, which is in line with a previous study focusing on splenic and Flt3L bone marrow–derived pDCs (Desai et al., 2007).

To study the effect of antigen uptake via activating and inhibitory FcγRs by DCs on the priming of CD4^+^ and CD8^+^ T cell responses, we chose an in vivo antigen-targeting approach by genetically fusing the antigen to FcγRIIB-, FcγRIIB/III-, and FcγRIV-specific antibodies. By using a specific endocytosis assay (Liu and Johnston, 2013; Reuter et al., 2015), we found that both activating and inhibitory FcγRs initiated antigen uptake into the conventional splenic DC subpopulations to a similar extent. As expected, internalization into Ly6C^high^ and Ly6C^low^ monocytes, but not NK, B, or T cells, could also be observed (Fig. 1 b). Functional antigen presentation, ultimately enabling T cell proliferation, was only observed for the cDC subsets (Figs. 5, 6, and 7), which may be explained by their superior capacity to transport antigens into MHC class I and MHC class II loading compartments (Ukkonen et al., 1986; Pierre and Mellman, 1998; Inaba et al., 2000; Turley et al., 2000; Delamarre et al., 2005; Trombetta and Mellman, 2005; Dudziak et al., 2007; Kamphorst et al., 2010). However, we cannot exclude that differential antigen trafficking is playing a role in individual DC subsets after antigen targeting, which may be addressed in future studies. All antigen-targeting antibodies induced a dose-dependent proliferation of antigen-specific CD4^+^ and CD8^+^ T cells (Fig. 2). Interestingly, antigen uptake and induction of T cell responses via activating FcγRIII and IV did not require ITAM signaling (Fig. 3, b and c). This is in contrast to the essential role of ITAM signaling for the induction of effector functions such as ADCC (de Haij et al., 2010; Boross et al., 2014). Interestingly, our further experiments revealed that regardless of whether antigen uptake occurred via inhibitory or activating FcγRs, the outcome after initial T cell proliferation was a rapid decline in T cell numbers under steady-state conditions (Fig. 3, d and e). In contrast, an enhanced and longer-lasting T cell response was observed under immune stimulatory conditions, when a broadly activating adjuvant, such as αCD40/pIC or HKLM, was applied (Fig. 3, d and e). In line with our previous results, CD4^+^ T cell responses were mostly initiated via CD11c^+^CD8^−^ DCs, whereas CD11c^+^CD8^+^ DCs efficiently induced CD8^+^ T cell proliferation (Fig. 7; Dudziak et al., 2007). Notably, FcγRIV was the only receptor that was able to induce strong CD4^+^ and CD8^+^ T cell responses in naive mice (Fig. 2; Fig 3, d and e; and Fig. 8). As this effect was of further interest, we also tested whether adding an αFcγRIV antibody without antigen might serve as a co-stimulus to foster naive CD4^+^ T cell responses induced by the other antigen-targeting antibodies. This was not the case, with the exception of a small but significant enhancement of the CD8^+^ T cell response induced by targeting of Ova to DEC205 (Fig. 8, a–d). By using an in vivo killing assay (Quah et al., 2012), we could demonstrate that antigen targeting to activating and inhibitory Fc receptors as well as to DEC205 and DCIR resulted in the generation of cytotoxic T cells (Fig. 8, e and f).

Collectively, our study shows that not only the type of FcγR (activating vs. inhibitory) but also the environment (steady state vs. proinflammatory) determines whether antigen uptake via FcγRs on DCs will result in short-term or long-lasting T cell responses. On a cellular level, the DC subset and not the type of receptor will govern whether a CD4^+^ or CD8^+^ T cell response will be triggered. With respect to the coexpression of activating and inhibitory FcγRs on both conventional splenic DC subsets, this may ensure an optimal T cell response via copriming of CD8^+^ and CD4^+^ T cell responses. Future studies using this strategy might help to elucidate the functional role of the various antigen-presenting cell populations, including DCs, in other lymphoid and nonlymphoid tissues. The capacity of Fc receptor targeting, particularly to FcγRIV or its human orthologue, FcγRIIIa, to induce strong and long-lasting CD4^+^ and CD8^+^ T cell responses may open new avenues to shape immune responses to self- and pathogen-derived antigens. With respect to immunotherapeutic approaches, this study may suggest that targeting antigens to FcγRs on DCs may be a promising approach to raise immunogenic or tolerogenic T cell responses to antigens of choice.

## Materials and methods

### Mice and cell lines

Female C57BL/6 mice were purchased from Charles River. TLR4^−/−^, CD11c-Cre, LysM-Cre, CD45.2^+^ OT-I, and CD45.2^+^ OT-II mice were obtained on C57BL/6 background from The Jackson Laboratory. OT-I and OT-II mice were crossed with CD45.1^+^ B6.SJL-Ptprc^a^Pep3^b^/BoyJ mice to a CD45.1 background. FcγRI^−/−^ (R1 B6), FcγRIIB^−/−^ (R2 B6), FcγRIII^−/−^ (R3 B6), FcγRIV^−/−^ (R4 B6), and FcγRIV^fl/fl^ mice were generated in the laboratory of Jeffrey Ravetch and are on the C57BL/6 background (The Rockefeller University). FcγRIV^fl/fl^ animals were crossed to LysM-Cre and CD11c-Cre mice to generate FcγRIV^fl/fl^ × LysM-Cre and FcγRIV^fl/fl^ × CD11c-Cre mice, respectively. NOTAM mice were generated in the laboratory of Jeanette Leusen on C57BL/6 background and backcrossed at least 15 times to C57BL/6 FcRγ^−/−^ mice (de Haij et al., 2010; Boross et al., 2014). Littermates were cohoused during all experiments. All mice were maintained under specific pathogen–free conditions. Generally, animals were included in experiments at the age of 6–12 wk. Protocols were performed according to institutional and national guidelines and evaluated by the Animal Welfare Committee of the local governmental authorities (Regierung von Mittelfranken, Ansbach and Regierung von Unterfranken Würzburg, Germany).

HEK293T cells were cultured in DMEM supplemented with 10% FCS, 100 U/ml penicillin, 100 µg/ml streptomycin, and 2 mM l-glutamine. Wild-type and stably with FcγRIIB or FcγRIV in combination with Fc receptor γ-chain–transfected CHO cells were cultured in supplemented RPMI1640 (5% FCS, 100 U/ml penicillin and 100 µg/ml streptomycin, 2 mM l-glutamine, and 1% nonessential amino acids). αFcγRIIB and αFcγRIV hybridoma cells were cultured in ISF-1 medium (Biochrom) supplemented with 100 U/ml penicillin and 100 µg/ml streptomycin.

### Isolation and preparation of primary cells

Single-cell suspensions from mouse spleen tissue were obtained by digestion with (FACS) or without (endocytosis assay, ex vivo proliferation assay) Collagenase D (Serva) and DNase I (Roche) as described before (Dudziak et al., 2007). Erythrocyte lysis was performed with ACK solution (Invitrogen), followed by filtering through a 40-µm mesh (BD) and washing in FACS (PBS, 2% FCS, and 0.09% NaN_3_) or MACS buffer (PBS and 0.5% BSA, degased).

### Antibodies used

If not otherwise stated, antibodies were purchased from eBioscience, BD, BioLegend, SouthernBiotech, or Invitrogen or produced in-house. Used antibodies were CD3ε (clone eBio500A2: eFluor450), CD4 (clone RM4-5: APC-eFluor780), CD8α (clone 53–6.7: APC-eFluor780, PerCP-Cy5.5), CD11b (clone M1/70: APC-e780, FITC, PE-Cy7), CD11c (clone N418: APC-eFluor780; clone HL3: FITC, PE), CD16 (FcγRIII; clone 275003: PE), CD16/32 (FcγRIIB/FcγRIII; clone 2.4G2: PE), CD19 (clone eBio1D3: eFluor450), CD25 (clone 3C7: APC), CD32 (FcγRIIB; clone K75.325: PE), CD40 (clone 3/23: PE), CD44 (clone IM7: APC, FITC), CD45R (B220; clone RA3-6B2: Alexa Fluor 488), CD45.1 (clone A20: eFluor450), CD49b (clone DX5: eFluor450, FITC), CD62L (MEL-14: PE), CD64 (FcγRI; clone 290322: PE, clone X54-5/7.1: PE, Alexa Fluor 647), CD69 (clone H1.2F3: PE-Cy7), CD80 (clone 16-10A1: FITC, PE), CD90.1 (clone HIS51: eFluor450), CD205 (DEC205; clone NLDC-145: Rockefeller monoclonal antibody facility; Alexa Fluor 647; clone 205yekta: eBioscience, PerCP-eFluor710), CD317 (PDCA-1; clone JF05-1C2.4.1: FITC, Alexa Fluor 647), CD335 (NKp46; clone 29A1.4: FITC, eFluor450), DCIR2 (clone 33D1: Rockefeller monoclonal antibody facility; Alexa Fluor 488, Alexa Fluor 647, PE), FcγRIV (clone 9E9: Rockefeller monoclonal antibody facility, Alexa Fluor 647, PE), INFγ (clone XMG1.2, Alexa Fluor 700, PE), Ly6C (clone AL21: APC; clone HK1.4: PE), Ly6G (clone 1A8: FITC, PacificOrange, PacificBlue, V450), MHC-II (clone AF6-120.1: Biotin, FITC, PerCP-Cy5.5), Siglec-H (clone eBio440C: FITC), TCR-Vα2 (clone B20.1: Biotin, PE). Secondary antibodies used were αmsIgG1 (goat F(ab′)_2_ α–mouse-IgG1 human adsorbed: PE), αArHamIgG (goat α–ArmenianHamster IgG: PE). Isotype controls used were ArHam-PE (clone eBio299Arm: PE), msIgG1 (clone MOPC-31C: PE; MOPC-21: Alexa Fluor 647), msIgG2a (clone MOPC-173: Alexa Fluor 647, PE), ratIgG2a (clone R35-95: Alexa Fluor 647, PE), ratIgG2b (clone A95-1: Alexa Fluor 647, PE). Other stains used were DAPI, PI, Strepavidin-PerCP-Cy5.5, Strepavidin-PE-Cy7, Strepavidin-V500, and FcγRIIB/III (clone 2.4G2; Bio X Cell), FcγRIIB (clone CT17.2; produced in-house), and FcγRIV (clone 9E9; produced in-house) for blocking.

### FACS analysis, direct and indirect endocytosis assay, and cell sorting

All FACS steps were performed in FACS buffer (PBS, 2% FCS, and 0.09% NaN_3_) on collagenase D/DNase I–treated single-cell suspensions. Where applicable, unwanted binding of FACS antibodies to Fc receptors was prevented by blocking of FcγRIV with the 9E9 blocking antibody in addition to blocking FcγRIIB and FcγRIII by 2.4G2 (Bio X Cell) for 15 min at 4°C. In the first staining step, cells were incubated with all directly labeled and biotinylated antibodies for 15 min at 4°C. After washing, biotinylated antibodies or unlabeled antibodies were visualized using conjugated Streptavidin or secondary antibodies for 15 min at 4°C, respectively. For the detection of FcγRs, first the specific FcγR antibody was stained combined with an excess of blocking antibodies for the remaining FcγRs (clone CT17.2 for FcγRIIB; Invitrogen and produced in-house; clone 275003 for FcγRIII; R&D Systems; and clone 9E9 for FcγRIV; produced in-house) for 15 min at 4°C. Afterward, all other antibodies were stained as described above.

Endocytosis assays were performed on directly isolated, Collagenase D (Serva)–, and DNase I (Roche)–treated single-cell suspensions from spleens. For the indirect endocytosis assay, splenocytes were stained with 2 µg/ml αFcγRIIB-Ova (clone CT-17.2, see below) or 1 µg/ml anti–FcγRIV-Ova (clone 9E9, see Cloning of Fc receptor–specific antibodies and Production and purification of targeting antibodies) after blockade of FcγRIV and FcγRIII (clone 275003; R&D Systems) or FcγRIIB and FcγRIII (clone 2.4G2; Bio X Cell), respectively, in FACS buffer for 20 min on ice. After intensive washing, cells were resuspended in RMPI1640 supplemented with 5% FCS, 2 mM l-glutamine, 100 U/ml penicillin, 100 µg/ml streptomycin, and 50 µM β-mercaptoethanol. Cells were incubated on 37°C (0 min, 120 min), instantly cooled down on ice, and directly fixed with 1% formaldehyde for 10 min on ice. Afterward, αmsIgG1-PE was applied as secondary antibody. After intensive washing, cells were stained with cell identification antibodies as described above.

To assess the uptake of the antibodies to directly compare the efficiency of the select receptors and the respective antibodies used for antigen targeting in this study, we applied a new labeling and quenching technique developed by Liu et al. (Liu and Johnston, 2013; Reuter et al., 2015). In brief, we functionally modified our targeting antibodies with a 10-fold molecular excess of DIBO-SE (2 nmol/µl in DMSO; Molecular Probes) in PBS for 2 h. After removing the excess of DIBO-SE by a ZEBA desalting column (MWCO 7 kD; Thermo Fisher Scientific), the antibodies were labeled with a twofold molecular excess of an Atto647N-5′-TCAGTTCAGGACCCTCGGCT-3′-N_3_ oligo (Biomers.net) for 16 h. The labeled antibodies were purified using a ZEBA desalting column (MWCO 40 kD; Thermo Fisher Scientific). To study their internalization, Collagenase D– and DNase I–treated splenic single-cell suspensions were incubated with the labeled antibodies in RMPI1640 supplemented with 5% FCS, 2 mM L glutamine, 100 U/ml penicillin, 100 µg/ml streptomycin, and 50 µM β-mercaptoethanol for 0, 10, 30, 60, or 120 min at 37°C. Extracellular fluorescence was quenched by adding an excess of a 5′-AGCCGAGGGTCCTGAACTGA-3′-BBQ650 oligo (Biomers.net). After washing, cells were stained with the cell identification antibodies as described before.

All FACS analyses were performed on a FACS-Canto II (BD) and LSR Fortessa (BD), and cell sorts were performed on a FACSAria II (BD) or MoFlo (Beckman Coulter) system. Data were analyzed with DIVA (BD) and FlowJo (Tree Star) Software.

### Cloning of Fc receptor–specific antibodies

Fc receptor antibodies αFcγRIIB (CT17.2), αFcγRIV (9E9), and αFcγRIIB/III (2.4G2) were cloned from the original hybridoma cells. RNA was prepared by QIAGEN RNeasy Mini kit and reverse transcribed into specific cDNA by using Superscript III (Invitrogen) and gene-specific primers (5′-GACAGGGATCCAGAGTT-3′ for αFcγRIV-HC, 5′-TAGAAGTCATTAACCAGACACACCA-3′ for αFcγRIV-LC, 5′-GACAGGGATCCAGAGTT-3′ for αFcγRIIB-HC, 5′-CCTGTTGAAGCTCTTGACA-3′ for αFcγRIIB-LC, 5′-ATTCCCGTAGTCTCTGTTGC-3′ for αFcγRIIB/III-HC, and 5′-GATGTCTCTGGGATAGAAGTT G-3′ for αFcγRIIB/III-LC) or an anchored dT_18_-Primer (5′-TTTTTTTTTTTTTTTTTTVN-3′). After tailing of the gene-specific cDNA by TdT (Thermo Fisher Scientific) with dG or dA (Roche), a PCR with a proofreading enzyme (Phusion, Phusion HS, or Phusion HS II, all Finnzymes) using a second nested gene specific primer (5′-GTACTCTAGAGGTCAAGGTCACTGGCTCA-3′ for αFcγRIV-HC, 5′-TTCGTAGTCTTCACCCCATCATTG-3′ for αFcγRIV-LC, 5′-GTACTCTAGAGGTCAAGGTCACTGGCTCA-3′ for αFcγRIIB-HC, 5′-GTACTCTAGAGGGTGAAGTTGATGTCTTGTC-3′ for αFcγRIIB-LC, 5′-ATTCCCGTAGTCTCTGTTGC-3′ for αFcγRIIB/III-HC, and 5′-ACAGTAATAGAGTCCAAAATCTTCAGG-3′ for αFcγRIIB/III-LC) and an abridged anchor primer (5′-CAGATCGGCCACGCGTCGACTAGTATTTTTTTTTTTTTTTTTTTTTVN-3′ or 5′-GGCCACGCGTCGACTAGTACGGGIIGGGIIGGGIIG-3′) was performed to amplify the DNA, which was then cloned into a blunt topoisomerase vector pSC-B-amp/kan (StrataClone Blunt PCR Cloning kit; Agilent Technologies). After sequencing, cloning product specific forward and reverse primers (5′-TCGTTTGAATTCGCCACCATGGCTGTCCTGGTGCTGCTG-3′ and 5′-AGATGGGGGTGTCGTTTTGGCTGAGGAGACGATGACCAGGGT-3′ for αFcγRIV-HC, 5′-TACCTTGAATTCGCCACCATGGCCTGGATTCCTCTCCTC-3′ and 5′-CTCACTGGATGGTGGAAACACTGTGACTTT-3′ for αFcγRIV-LC, 5′-ACGATCGAATTCGCCACCATGAAATTCAGCTGGGTCATCTTC-3′ and 5′-CAGGGGCCAGTGGATAGACCGATGG-3′ for αFcγRIIB-HC as well as 5′-TAGTACGAATTCGCCACCATGGAGACAGACACAATCCTGC-3′ and 5′-GAGGCACCTCCAGATGTTAACTGCTCAC-3′ for αFcγRIIB-LC, 5′-GACTTTGAATTCGCCACCATGGACATCAGGCTCAGCTTG-3′ and 5′-TACTTTGCTAGCTTTACCCGGAGGCCGGGAGATGCTC-3′ for αFcγRIIB/III-HC, and 5′-TAACTTGAATTCGCCACCATGTCAGGACACAATTTAGATATGAGGG-3′, 5′-ACTCTCCAATCTGGCATCCCCAGCAGGTTCA-3′, 5′-TGAACCTGCTGGGGATGCCAGATTGGAGAGT-3′, and 5′-TACCTTGCTAGCACACTCATTCCTGTTGAAGCTCTTGA-3′ for αFcγRIIB/III-LC) were designed to amplify the variable regions of the HCs and light chains (LCs) from the whole cDNA pool. Overlap PCRs were used to generate fusion constructs of the original variable regions and a mutated (N297A) mouse IgG1 constant region (Hawiger et al., 2001; Dudziak et al., 2007), which inhibits binding of the antibodies to other Fc receptors via the antibody HC (5′-ACCCTGGTCATCGTCTCCTCAGCCAAAACGACACCCCCATCT-3′ for αFcγRIV-HC, 5′-AAAGTCACAGTGTTTCCACCATCCAGTGAG-3′ for αFcγRIV-LC, 5′-CCATCGGTCTATCCACTGGCCCCTG-3′ for αFcγRIIB-HC, 5′-CATCTGGAGGTGCCTCAGTCGTGTGCTT-3′ for αFcγRIIB-LC, 5′-GTCACTGTCTCCTCAGCCAAAACGACACCC-3′ and 5′-GGGTGTCGTTTTGCCTGAGAAGACAGTGAC-3′ for αFcγRIIB/III-HC, as well as 5′-CCCCGGGCTAGCTTTACCAGGAGAGTGGGAG-3′ for all HCs and 5′-CCCCGGGCGGCCGCTCAACACTCATTCC-3′ for all LCs). Chimeric antibody HCs were fused by restriction to an Ova open-reading frame (Dudziak et al., 2007) to generate the desired targeting antibodies αFcγRIIB-Ova, αFcγRIV-Ova, and αFcγRIIB/III.

### Production and purification of targeting antibodies

Chimeric antibodies were produced by transient transfection of HEK293T cells as described before (Hawiger et al., 2001; Dudziak et al., 2007). In brief, plasmids containing antibody LC and HC were transfected by calcium phosphate precipitation, and supernatants were collected 1 wk later and concentrated by ammonium sulfate precipitation. Antibodies were purified by incubation with protein G Sepharose beads (GE Healthcare). Endotoxin was removed by treatment with Triton X-114 and afterward examined by LAL test (Lonza). Antibodies were only used (valid for all performed assays) when the endotoxin level was <0.001 EU/µg.

SDS-PAGE was performed as described before (Hawiger et al., 2001; Dudziak et al., 2007). In brief, loading dye with dithiothreitol was added to the protein solutions and heated to 95°C for 10 min and then put on ice. Afterward, proteins were run in 10% or 12.5% SDS-polyacrylamide gels. Gels were stained in Coomassie R-250 solution and destained in an acetic acid/methanol mixture (Hawiger et al., 2001; Dudziak et al., 2007; Heidkamp et al., 2010).

### In vivo proliferation assay

Ova-specific transgenic T cells were isolated from CD45.1^+^ OT-I or CD45.1^+^ OT-II mice by positive selection of CD8^+^ or CD4^+^ T cells using paramagnetic beads (Miltenyi Biotec). Purity (≥95%) was assessed regularly by flow cytometry. After CFSE labeling (Thermo Fisher Scientific), 10^6^ CD8^+^ OT-I or 2 × 10^6^ CD4^+^ OT-II T cells were i.v. injected into congenic CD45.2^+^ recipient mice. 16 h later, targeting antibodies were i.p. injected in different amounts as indicated in the experiments. Mice were sacrificed, and splenocytes were analyzed for proliferation of the transferred transgenic T cells. In case of depletion of specific cell populations, mice were i.v. injected with PBS or 20 ng/g body weight of DT (Merck or Sigma-Aldrich) 24 h before antibody injection. For depletion of pDCs, 200 µg αPDCA-1 antibody was i.p. injected 72, 48, and 24 h before injection of the targeting antibodies.

### Ex vivo proliferation assay

C57BL/6 mice were i.p. injected with 10 µg (or 30 µg) αDEC205-Ova, αDCIR2-Ova, αFcγRIIB-Ova (30 µg), αFcγRIV-Ova, or 30 µg isotype-Ova control antibodies. 12 h later, mice were sacrificed, and single-cell suspensions from spleens were positively enriched for a mixture of CD11c^+^ and CD115^+^ cells, as well as CD19^+^ B cells by MACS beads (Miltenyi Biotec). CD11c^+^CD115^+^ cells were further separated by cell sorting on a FACS-Aria-II or a MoFlo system (Beckman Coulter) into lin^−^ (CD3^−^CD19^−^NKp46^−^Ly6G^−^Siglec-H^−^) CD11b^high^CD11c^low^Ly6C^high^, lin^−^CD11b^high^CD11c^int^ Ly6C^low^, lin^−^CD11b^neg^CD11c^high^CD8^+^, and lin^−^CD11b^pos^CD11c^high^CD8^−^ cells. Cell sort purified cells and CD19^+^ MACS bead–enriched B cells were irradiated (3 Gy) and co-cultured with positive MACS bead–enriched CD8^+^ OT-I or CD4^+^ OT-II T cells. 16 h (OT-I) or 40 h (OT-II) later, ^3^H-thymidine was added to the cultures. T cell proliferation was assessed by measurement of ^3^H-thymidine incorporation 24 h later.

### Restimulation assays

The whole assay was performed in RPMI1640 medium supplemented with 2 mM l-glutamine, 5% heat-inactivated FCS (Biochrom), 50 U/ml penicillin, 100 µg/ml streptomycin, and 50 µM β-mercaptoethanol in 48-well plates. C57BL/6 mice were i.p. injected with 10 µg αDEC205-Ova, αDCIR2-Ova, αFcγRIIB-Ova, αFcγRIV-Ova, αFcγRIIB/III-Ova, or isotype-Ova in the presence of 50 µg αCD40 antibody (clone 1C10) and 25 µg poly(I:C) (InvivoGen) or 10^9^ HKLM. 14 d later, 10^5^ freshly isolated MACS bead–enriched CD11c^+^ cells from naive C57BL/6 animals were loaded with an overlapping Ova peptide pool (Miltenyi Biotec) according to the manufacturers’ instructions, 2 µM peptide 75 (EKLTEWTSSNVMEER-OH, containing a CD8^+^ and a CD4^+^ T cell epitope; Boscardin et al., 2006), or incubated in medium alone. 2 × 10^6^ splenocytes from antigen-targeted animals were added together with 2 µg/ml co-stimulatory αCD28 antibody (clone 37.51), and 2 h later, 5 µg/ml cytokine-trapping Brefeldin A (BD) was added. A stimulation with 1 µg/ml αCD3 antibody and 2 µg/ml αCD28 antibody served as a positive control and stimulation with αCD28 antibody alone as a negative control. After 8 h, cells were washed and subjected to intracellular cytokine staining. For this purpose, cells were fixed and permeabilized according to the manufacturer’s protocol by Perm/Fix and Perm/Wash (Cytofix/Cytoperm kit; BD), respectively. Cells were stained with antibodies for B220, CD4, CD8, and IFNγ.

### Preparation of target cell mixture for in vivo killing assays

We isolated splenocytes of congenic CD45.1^+^ B6.SJL-PtprcaPep3b/BoyJ mice and generated 10 differentially labeled cell populations. Therefore, we first labeled the cells with two different concentrations of CFSE (3 µM or 15 µM) for 20 min, 37°C in the dark as described before (Quah et al., 2012). After washing the cells in FCS containing RPMI medium, we further labeled these two CFSE-marked populations with each five different concentrations of cell trace violet (0 µM, 0.3 µM, 1.5 µM, 7.5 µM, or 37.5 µM) for 20 min, 37°C in the dark to generate altogether 10 easy-to-distinguish target populations (3 µM::0 µM, 3 µM::0.3 µM, 3 µM::1.5 µM, 3 µM::7.5 µM, 3 µM::37.5 µM, 15 µM::0 µM, 15 µM::0.3 µM, 15 µM::1.5 µM, 15 µM::7.5 µM, and 15 µM::37.5 µM). These 10 populations were finally loaded with different concentrations of SIINFEKL peptide (3 µM::0 µM w/o peptide, 3 µM::0.3 µM with 2.4 nM SIINFEKL, 3 µM::1.5 µM with 40 nM SIINFEKL, 3 µM::7.5 µM with 160 nM SIINFEKL, 3 µM::37.5 µM with 625 nM SIINFEKL, 15 µM::0 µM with 625 nM SIINFEKL, 15 µM::0.3 µM with 160 nM SIINFEKL, 15 µM::1.5 µM with 40 nM SIINFEKL, 15 µM::7.5 µM with 2.4 nM SIINFEKL, and 15 µM::37.5 µM w/o peptide). After intensive washing, these different populations were mixed in a 1:1:1:1:1:1:1:1:1:1 ratio in PBS.

### In vivo killing assays

Naive C57BL/6 mice were immunized i.p. with 3 µg of the targeting antibodies (αDEC205-Ova, αDCIR2-Ova, αFcγRIIB-Ova, αFcγRIV-Ova, αFcγRIIB/III-Ova, or isotype-Ova) in PBS with 50 µg αCD40 antibody (clone 1C10) and 25 µg poly(I:C) (InvivoGen). Eight days later, mice were challenged with a mixture of the prepared target cells by i.v. injection of 10^7^ cells. 16 h later, splenocytes were reisolated and stained for CD45.1 and DAPI and analyzed for the number of remaining cells of the different transferred SIINFEKL-loaded and control populations (0 nM, 2.4 nM, 40 nM, 160 nM, and 625 nM). Specific lysis was calculated by using two different non-SIINFEKL-loaded populations accounting for injection variances and unspecific lysis of the target cells (Quah et al., 2012).

### HKLM

To prepare the HKLM, a bacterial culture of *L. monocytogenes* 10403S was harvested in late log phase, centrifuged, and washed three times in PBS. The recovered bacteria were resuspended in PBS and incubated at 70°C for 20 min. Bacterial concentration before killing and absence of viable bacteria after heating were monitored by counting the CFU on brain–heart infusion agar plate. The 1.0 × 10^10^ HKLM/ml suspension was stored at −80°C.

### Online supplemental material

Fig. S1 shows the gating strategy of DC subpopulations and other cells in mouse spleen. Fig. S2 shows depletion of cDCs by DT injection in CD11c-DTR transgenic mice and pDC depletion by αPDCA-1 injection in C57BL/6 mice.

## Supplementary Material

Supplemental Materials (PDF)
