# Supplementary material for: DC subset–specific induction of T cell responses upon antigen uptake via Fcγ receptors in vivo
Source: J Exp Med. 2017 May 1;214(5):1509–28. doi: 10.1084/jem.20160951 (PMC5413326; doi:10.1084/jem.20160951)
Supplement: Supplemental Materials (PDF) [file JEM_20160951_sm.pdf]

SUPPLEMENTAL MATERIAL

Lehmann et al., https://doi.org/10.1084/jem.20160951

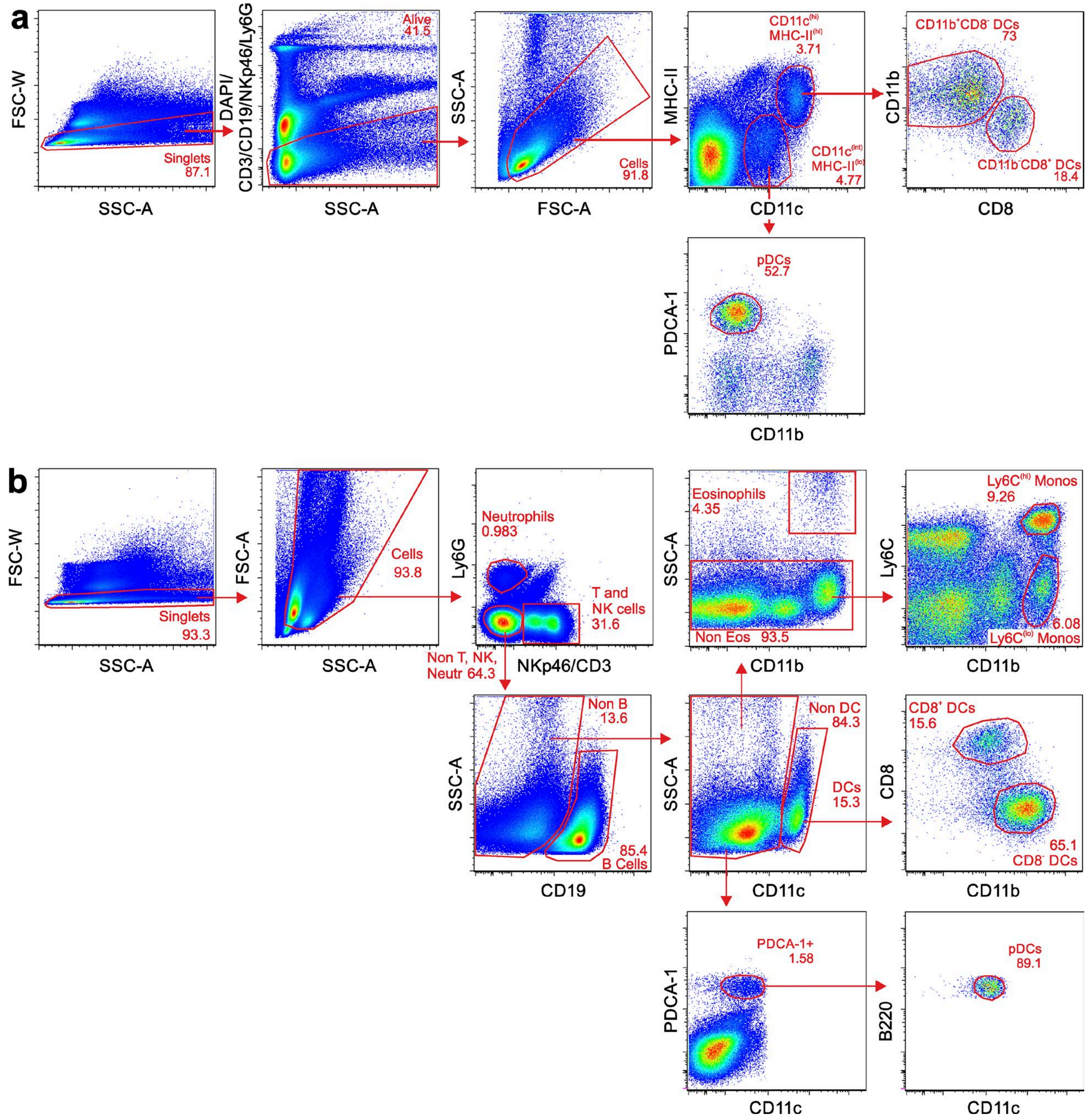

Figure S1. **Gating strategy of DC subpopulations and other cells in mouse spleen.** (a) Spleen single-cell suspensions were generated from C57BL/6 and the respective FcγR knockout mice by Collagenase D/DNase I digestion. Cells were stained with DAPI, CD11c, PDCA-1, CD11b CD8, MHC-II, Ly6G, CD3, CD19, CD335 (NKp46), and the corresponding FcγR antibodies. In all experiments, doublets were excluded by SSC-A/FSC-W gating followed by exclusion of T cells, B cells, NK cells, and neutrophils (lin$^-$: CD3$^-$CD19$^-$Ly6G$^-$NKp46$^-$). (a) CD11c$^+$ cells were further analyzed for CD11c^{high}MHC-II^{high}CD11b$^+$CD8$^-$ DCs (CD11b$^+$CD8$^-$ DCs), CD11c^{high}MHC-II^{high}CD11b$^-$CD8$^+$ DCs (CD11b$^-$CD8$^+$ DCs), and CD11c^{low}MHC-II^{low}CD11b$^-$PDCA1$^+$ pDCs (pDCs). (b) For analysis of recombinant antibody uptake, whole splenocytes were isolated, labeled with the recombinant Ova-carrying antibodies, and incubated for 120 min at 37°C or on ice, respectively. After fixation, cells were stained with the secondary and cell identification antibodies αmsIgG1, CD3, CD8, CD11b, CD11c, CD19, CD45R (B220), CD335 (NKp46), Ly6C, Ly6G, and PDCA-1. Pseudocolor dot plots show doublet exclusion by SSC-A/FSC-W gating followed by exclusion of debris and dead cells with gates set as Ly6G$^+$ (neutrophils), NKp46$^+$CD3$^+$ (T and NK cells), and Ly6G$^-$NKp46$^-$CD3$^-$ cells. From the latter ones, CD19$^+$ cells (B cells) were further gated in a SSC-A/CD19 blot. CD19$^-$ cells were depicted in a SSC-A/CD11c blot, in which CD11c^{high} cells were analyzed for CD8 (CD11c$^+$CD8$^+$) and CD11b (CD11c$^+$CD8$^-$) expression. From the SSC-A/CD11c$^-$ gate, cells were further analyzed for CD11b expression in a SSC-A/CD11b gate. SSC-A^{high}/CD11b^{high} cells characterize eosinophils. All SSC-A^{low} cells were split up into Ly6C^{high}CD11b^{high} and Ly6C^{low}CD11b^{high} cells. pDCs were gated as CD19$^-$, CD11c^{low}, PDCA-1$^+$, and B220^{low}. FACS analysis was performed on a FACSCanto II, and data were analyzed using DIVA and FlowJo software. The experiment was repeated at least three times with similar results.

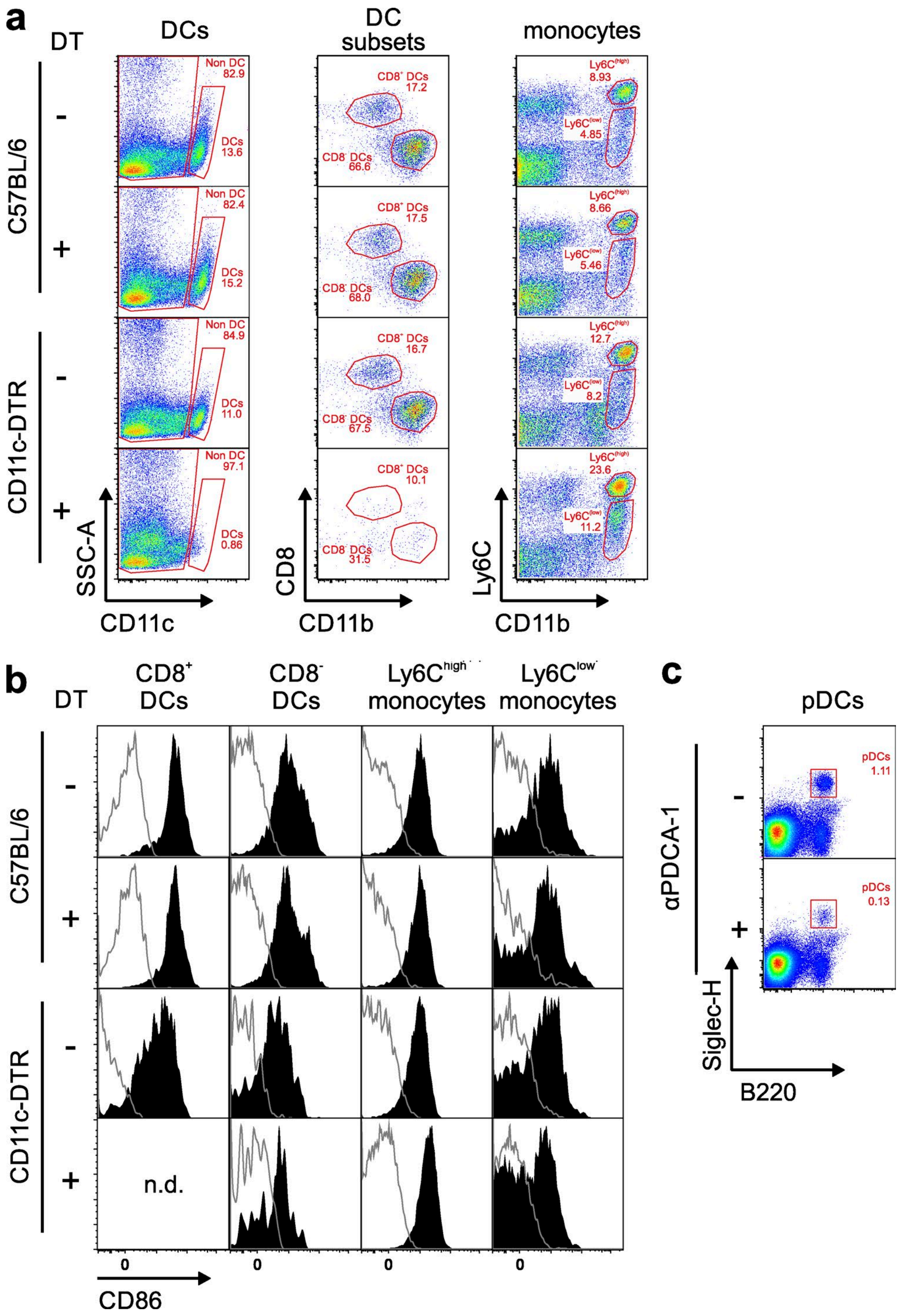

Figure S2. **Depletion of cDCs by DT injection in CD11c-DTR transgenic mice, and pDC depletion by αPDCA-1 injection in C57BL/6 mice.** CD11c-DTR transgenic and C57BL/6 mice were treated i.p. with 20 ng/g bodyweight DT or PBS. (a and b) 24 h later, splenocytes were reisolated and analyzed for DC and monocyte subpopulations (a) and their activation status (b; gating was performed as shown in Fig. S1). n.d., not detected. (c) C57BL/6 mice were i.p. treated three times (72, 48, and 24 h before analysis) with 200 µg αPDCA-1 antibody. Splenic single-cell suspensions were generated, and Siglec-H$^+$B220$^+$ pDC numbers were analyzed by FACS. The experiment was repeated three times with similar results.
